# From Bernstein's rheotome to Neher‐Sakmann’s patch electrode. The action potential

**DOI:** 10.14814/phy2.13861

**Published:** 2019-01-03

**Authors:** Edward Carmeliet

**Affiliations:** ^1^ Katholieke Universiteit Leuven Leuven Belgium

**Keywords:** Conduction, ionic theory, pacemaker, patch

## Abstract

The aim of this review was to provide an overview of the most important stages in the development of cellular electrophysiology. The period covered starts with Bernstein's formulation of the membrane hypothesis and the measurement of the nerve and muscle action potential. Technical innovations make discoveries possible. This was the case with the use of the squid giant axon, allowing the insertion of “large” intracellular electrodes and derivation of transmembrane potentials. Application of the newly developed voltage clamp method for measuring ionic currents, resulted in the formulation of the ionic theory. At the same time transmembrane measurements were made possible in smaller cells by the introduction of the microelectrode. An improvement of this electrode was the next major (r)evolution. The patch electrode made it possible to descend to the molecular level and record single ionic channel activity. The patch technique has been proven to be exceptionally versatile. In its whole‐cell configuration it was the solution to measure voltage clamp currents in small cells.

**See also:** https://doi.org/10.14814/phy2.13860 & https://doi.org/10.14814/phy2.13862

## Bernstein and Membrane Hypothesis

My overview starts in the middle of the 19th century with three important observations. (1) Carlo Matteuci, 1842 measured an injury current between the cut and the intact surface of nerve or muscle; (2) Emil du Bois‐Reymond, 1848, showed that this injury current decreased during repetitive stimulation; the decrease was called the negative variation; (3) Hermann von Helmholtz, 1849 measured the conduction velocity of the nerve impulse at 30 m/sec. This start was successfully continued by Julius Bernstein (1839–1919), who as a postdoctorate stayed first with Emil du Bois‐Reymond in Berlin and continued afterward with Hermann von Helmholtz in Heidelberg.

### Membrane theory

In 1872 Bernstein was promoted professor in Halle. Being from Jewish origin he could, strictly spoken, not be nominated at the protestant Martin Luther University. But he obtained dispensation. A generation later in the 1930s, his two sons also university professors, got a different treatment. On a trip to the United States one of them got the news that he better could stay in the United States than coming back to Germany. The other son also fled from Germany.

**Figure 1:**
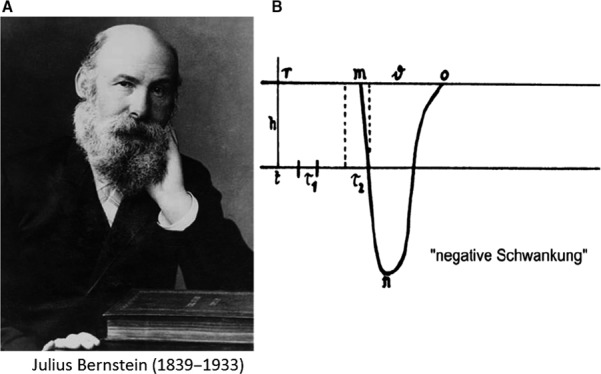
(A) Photograph of Julius Bernstein at the time of his rectorship at the University of Halle (1890). With permission reproduced from (Seyfarth 2006). (B) Drawing of the first action potential in nerve. The negative variation was measured using the rheotome build by Bernstein. With permission reproduced from (Nilius 2003).

Julius Bernstein was nine times dean of the faculty of medicine and 1 year (1890) rector of the University (the picture in Fig. 1A dates from that period). Bernstein's most important achievement is the formulation of the Membrane Hypothesis. He worked on it during the second half of the 19th century and published two important volumes, Untersuchungen zur Thermodynamik der bioelektrischen Ströme, in 1902(Bernstein 1902) and Elektrobiologie, Die Lehre von den elektrischen Vorgängen im Organismus auf moderner Grundlagen dargestelt, in 1912 (Bernstein 1912). According to the Membrane hypothesis, the cell is surrounded by a membrane selectively permeable to K^+^ ions. Since the intracellular K^+^ concentration is larger than the extracellular concentration, K^+^ ions move outward and generate a negative intracellular potential. Bernstein used the thermodynamic analysis for diffusion potentials developed by Nernst (1864–1941) (Nernst 1889) to express the result by the equation:

$$V=\frac{\mathrm{RT}}{\mathrm{zF}}\text{In}\frac{{\left[K^{+}\right]}_{o}}{{\left[K^{+}\right]}_{i}}$$

In an intact fiber an electrical double layer is thus formed across the membrane. When injured, a short‐circuit is generated between the cut and the intact surface: the injury current. Upon cathodic stimulation the membrane loses its selective permeability to K^+^ ions and becomes permeable to all ions: the injury current drops to zero, this is the negative variation, or what we presently call the action potential. Because of the shift to nonselective permeability the action potential was assumed to show no overshoot. Propagation of the impulse was hypothetically proposed to occur via the mechanism of local currents. Ludimar Hermann, former assistant of du Bois‐Reymond in Berlin was a fervent defender of this theory. The electrical nature of the impulse propagation via local currents was far from generally accepted.

### Differential Rheotome. Negative variation

**Figure 2:**
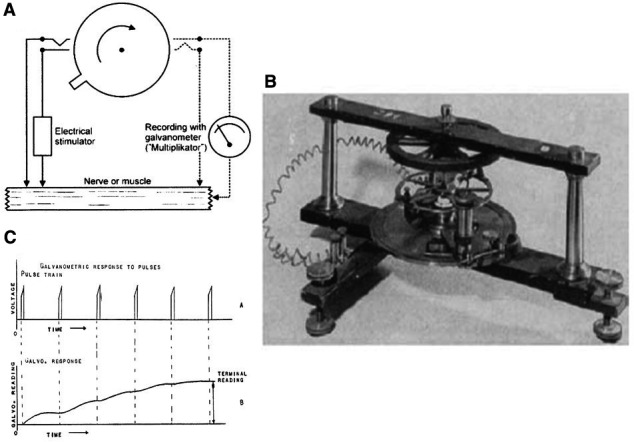
(A) Schematic of the Bernstein's rheotome (Seyfarth 2006). (B) Photograph of the real instrument (width 20 cm) build in Heidelberg circa 1870 according to (Nilius 2003). (C) Using very short sampling intervals the rheotome due to its inertia acts as integrator (Hoff and Geddes 1957). Reproduced with permission.

A second major achievement of Julius Bernstein was the construction in 1868 of a differential rheotome or “current slicer” (Hoff and Geddes 1957) (Fig. 2). Bernstein's aim was to obtain an accurate recording of the action potential time course. From experiments on the nerve‐muscle preparation and its response to elevated frequency stimulation it was expected to be a fast and short phenomenon (order of ms). Available instruments for measurement of electrical events at that time were galvanometers. But a galvanometer was and some are still slow in response (time constant order of seconds). Bernstein solved the problem of recording a fast phenomenon with a slow‐response instrument using the rheotome in a special configuration. The rheotome consists of three components: a turntable (diameter 20 cm) with a cam, a stimulator circuit (the Ruhmkorff induction coil) and a recording circuit with galvanometer (Fig. 2A). A photograph of a real specimen of the turntable is given in Figure 2B. Width of the instrument is 20 cm. When the turntable is moving clockwise the stimulator circuit is first activated followed after a given delay by activation of the recording circuit during a predetermined time, the sampling interval. The stimulus was repeated a number of times at 5–10/per sec. Under these circumstances the slow galvanometer, due to its inertia, acts as an integrator (Fig. 2C). After obtaining a reading, the delay was changed in order to cover the whole duration of the negative variation (Fig. 1B). Bernstein's findings using this instrument were as follows: (1) the negative variation or action potential is a short transient event (order of 1 msec) consisting of two fast phases; a first phase or depolarization during which the resting or injury potential disappears and a second phase or repolarization with a somewhat slower tail; (2) in nerve, Bernstein recorded an overshoot, clearly present. This observation was not stressed by him because he suspected the existence of a junction potential and his membrane hypothesis predicted no overshoot. The observation of an inversion of polarity, however, was later proven to be correct, (3) the negative variation in muscle is practically over before onset of contraction, (4) the rate of propagation is 28.7 m/sec. This was indeed the first objective and accurate measurement of a nerve action potential. The major technical progress made by Bernstein consisted of changing the response time for measuring a transient electrical phenomenon from tens of seconds to 10^−4^ sec. The time taken and the patience required to perform such a reading, however, were not negligible and further attempts of technical improvement for measuring the negative variation were initiated: the first was by Gabriel Lippmann with his electrometer in 1873. It was the type of instrument used by Augustus Waller to derive in 1887 the first human electrocardiogram, then still called electrogram. Willem Einthoven in his first years continued to use the electrometer and was able to describe the five different waves in the human electrocardiogram: P,Q,R,S,T, still used in the actual analysis of the electrocardiogram. Beginning 20th century Einthoven started the development of the string galvanometer and published in 1902 the first electrocardiogram pictures obtained using this new instrument. Instrumental and technical improvements did not stop and were made possible thanks to the production of the first vacuum tube in 1875 by William Crookes and the first cathode ray tube in 1897 by Karl Ferdinand Braun, still called the Braun tube.

### Membrane models

Although Bernstein formulated a clear picture about the function of the cell membrane, the chemical composition and the structural components of membranes remained a mystery. Around the turn of the century Meyer (1899, in Marburg) and Overton (1901, British scientist, distinct cousin to Charles Darwin and living in Zürich, later in Würzburg and Lund) performed a series of interesting experiments on the permeability of the cell membrane to anesthetics (Meyer 1899; Overton 1901). They found independently that the greater the partition coefficient oil/water of an anesthetic, the greater the coefficient of permeability through the membrane and the greater the potency of narcosis. They concluded that lipoids had to be an essential component of the cell membrane.

**Figure 3:**
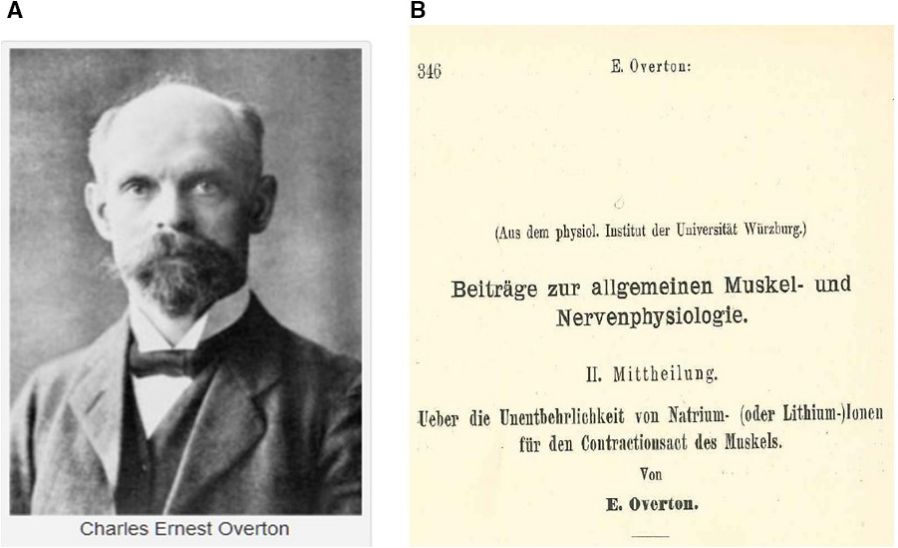
(A) Ernest Overton (1865–1933) known for his contributions on membrane permeability to lipid‐soluble anesthetics and for the study of ionic effects on membrane excitability. (B) title page of the study on the indispensability of sodium‐(or lithium) ions for the muscle contraction (Overton, 1902). Reproduced with permission.

This image of the membrane underwent further improvements. From an electrical point of view the presence of lipids predicted the existence of a capacity. In 1925 Fricke (Fricke and Morse 1925) working on erythrocytes measured a membrane capacity of 0.81 *μ*F/cm^2^. With a dielectric constant of 3 (apparently an underestimation) he calculated a transmembrane thickness of 3.3 nm, implying a monomolecular structure. After finding that the extracted lipids from erythrocytes had a surface in monolayer of twice the surface of the erythrocytes (Gorter and Grendel 1925) this estimate was corrected to a bilayer structure. The second chemical component, the globular proteins made their entry with Danielli (Danielli 1935). The picture was completed much later in 1972 by the fluid mosaic model of Singer. Overton (1865–1933) (Fig. 3A) was already mentioned for his work on the role of lipids in the membrane permeability to anesthetics (Overton 1901). He performed, however, also important investigations on other aspects of the membrane function. In 1902 he demonstrated that sodium (or lithium) were indispensable to elicit muscle contraction (Fig. 3B). He claimed that upon application of an electrical pulse the cell membrane became permeable to sodium and since it was known that the Na^+^ concentration in the cell cytoplasm was rather low he also predicted the necessity of a mechanism, which we now call the Na^+^‐K^+^ pump. These conclusions were based on the finding that a gastrocnemius‐nervus sciaticus preparation became electrically inexcitable in a sucrose (or glucose) ion‐free solution. It was the presence of Na^+^ (or Li^+^) as cation which was required for a normal excitability; the anion of the salt had no importance. Overton hypothesized that Na^+^ exchanged with K^+^ during the short latency between stimulus and the start of contraction, which, as we know presently, corresponds to the time the action potential is spread over the muscle surface. This period had to be short because otherwise the cell would gain too much sodium. And, since living cells were not rich in intracellular sodium, this sodium had to exported afterward with loss of energy. Overton used here the principle of minimum energy and in fact described what happens during a nerve or skeletal muscle action potential. In describing the necessity of sodium ions (and he rightfully added lithium ions) for a normal excitability and hypothesizing that external sodium exchanged with internal potassium Overton described the essence of what later Hodgkin, Huxley, and Katz (Hodgkin and Katz 1949) would demonstrate as the ionic theory.

## The Squid Giant Axon

Advance in scientific research depends heavily on occasional developments in technique, invention of a new technique, construction of a new instrument, using of a new preparation. The latter was the case with the introduction of the squid giant axon by John Z. Young in electrophysiology (1936).

**Figure 4:**
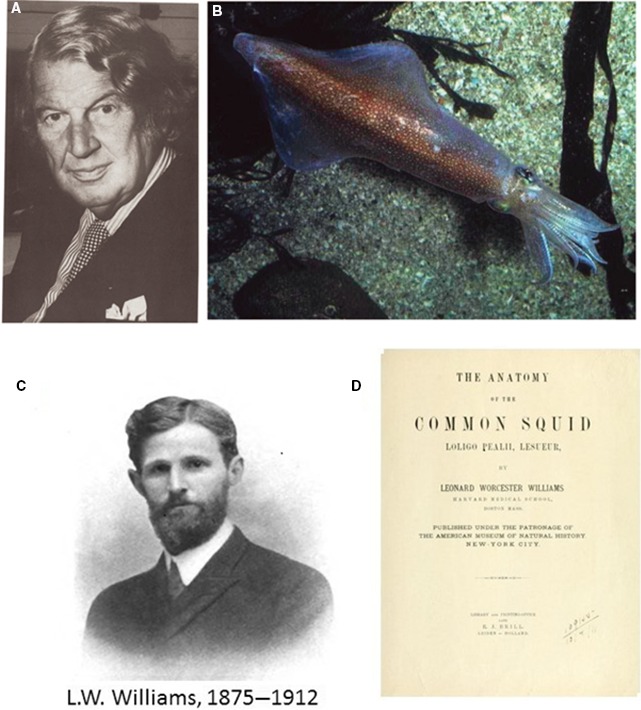
(A) J.Z.Young (1907–1997), professor of Anatomy University College London. Was the enthusiastic promotor of the use of giant axons of the squid and cuttlefish by neurophysiologists. (B) photo of Loligo forbesi. Reproductions from (Schwiening 2012) with permission. (C) L.W. Williams (1875–1912), professor of Anatomy at Harvard Medical School, started studies on the anatomy of the squid but died prematurely in an elevator accident at Harvard in 1912. (D) frontispiece of Williams’ thesis on the anatomy of the common squid. Photograph of Williams reproduced from Kingsley (1913), with permission.

John Z. Young (1907–1997) (Fig. 4A) was a British zoologist, who upon graduation obtained a scholarship to perform research on cephalopods in the Napoli Stazione Zoologica. His first papers appeared in 1929, but he returned regularly to Napoli and more than 150 papers and several books were published in that field. In 1934 he described in squid and cuttlefish the presence of large transparent tubes of 0.5 to 1.0 mm diameter, starting in the head and going down to the tail. Young presumed that these structures were axons. Histologically these tubes were syncytia and originated each from numerous, between 300 and 1500, small neurons.

Young was convinced that these structures were giant axons and could be of enormous help to electrophysiologists. In 1936 he went to Woods Hole U.S. Marine Biological Station to convince his physiology colleagues that these curious structures were indeed nerve fibers. He could demonstrate that stimulation of the giant axons caused a coordinated contraction of the mantle muscle and expulsion of a powerful water or ink jet, used in vivo to move and escape. Escape reactions are also of importance for fish and amphibia. Interesting to mention is that these species also use large diameter axons, the Mauthner cells, in escape reactions. John K. Young had no problems convincing Alan Hodgkin, GB, Cambridge and Kenneth Cole, United States, Columbia College, who were going to be competitors in the scientific race to the ionic theory of the action potential.

Honesty commands to mention that an American scientist L. W. Williams (1875–1912) (Fig. 4C) had already published on these peculiar anatomical structures of the squid in 1909, but without detailed anatomical description or hint for a possible function. Apparently there was no need in the electrophysiological community for this kind of preparation at that time. In 1912, 3 years after publication of his monograph, (Fig. 4D) (“The anatomy of the common squid Loligo pealii,” Lesueur. Publ. E.J.Brill, Leiden, Holland, 1909) Williams died prematurely in an elevator accident at Harvard Medical School and the work was forgotten for 25 years.

His widow, Martha Clarke, to get financial support for the education of their two children rented part of the house to Harvard students. It is a turn of fate that Kenneth Cole, competitor of Alan Hodgkin, but the first in line to voltage clamp the giant axon, boarded at the Martha Clarke's house and was able to read Williams’ thesis on the squid before meeting Young.

The monograph is available on internet and I have taken the opportunity to thumb through it. The work does not contain a complete anatomical description of the nervous system of the squid but on the other hand it is clear that Williams was convinced that exceptional large fibers were present in the squid and that they were of nervous origin. In a description of the two critic nerves he writes (p72): “The fibres of this nerve are unusually large and stain differently so that they can be readily traced for some distance in the ganglion.” And on p74: “A pair of cells, the two largest and most remarkable cells of the body are situated in the pedal ganglion…. The cytoplasm is granular and, like the very large nerve process arising from it, stains differently with haemalum. These fibres, not previously described in any mollusc, resemble closely the fibres of Mauthner in vertebrates, which are also unique in the nervous system of the animal….The very size of the nerve processes has prevented their discovery, since it is well‐nigh impossible to believe that such a large structure can be a nerve fibre.”

It is remarkable that Williams makes the comparison of the large fibers in the squid with the Mauthner fibers in vertebrates. Later Young will accentuate that in the squid as well as in the vertebrates the large fibers are used in escape reactions, just because of their high conduction velocity due to their large diameter.

As said this nice piece of work was forgotten for 25 years. In the summer of 1936, Young was in Woods Hole promoting the use of the squid giant axon. He was telling Cole: “If you want to find out about nerve, you've got to work on this axon.” During this conversation Cole asked the question how everyone had missed this half‐mm tube as an axon. Young said he had not done the literature until he'd mostly finished at Naples and then had found a 1912 monograph–by an American–on the giant axon of the squid. “Would that American be L.W.Williams?” Cole asked and told Young the story of his landlady's husband…to Young's utter amazement.

## Genesis of the Ionic Theory

### The prewar period

After the summer of 1936 in Woods Hole K.Cole and A.Hodgkin returned home and oriented their scientific research direction squid giant axon.

Alan Hodgkin (Fig. 7) had obtained his undergraduate education in Trinity College Cambridge in 1932–1935 after winning an open scholarship. At the start he hesitated between physiology and zoology. He decided to study physiology on the advice of one of his professors, that “all what experimental zoologists do is repeat on many animals what physiologists have demonstrated in one animal.” During his postgraduate year he obtained a research fellowship at Trinity College Cambridge and in 1937–1938 spent a year in New York Rockefeller University with Herbert Gasser. It was then that Alan Hodgkin learned from Kenneth Cole how to dissect and clean a giant axon. He became friends with him and with Howard Curtis at Columbia.

**Figure 5:**
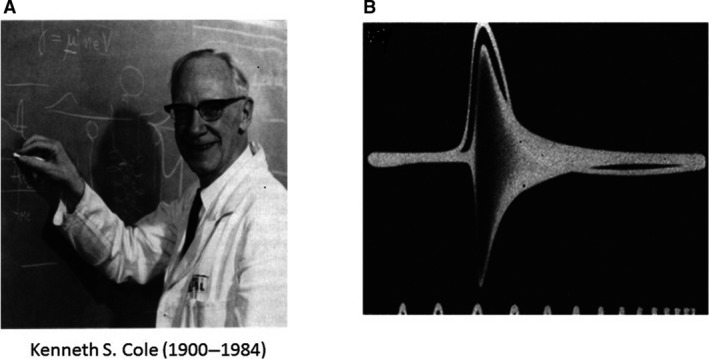
(A) K.S. Cole, physicist by training but performed many experiments with a physiological impact. He shifted definitively in the biological direction when he was promoted Professor of Physiology at the Columbia College of Physicians and Surgeons. Reproduced from (Goldman 1985) with permission. (B) His first experiment using the squid giant axon was to measure the impedance change during the action potential. Reproduced from (Cole and Curtis 1949) with permission. The impedance measurement was not only a scientific success but had also an artistic dimension. The picture was taken by the Biophysics section of the Biophysical Society as their logo. In Sweden it appeared in modern apartments in a slightly modified form with the time axis and the impedance axis exchanged. The result was highly appreciated.

Kenneth S. Cole (1900–1984) (Fig. 5A) was a physicist of training; he obtained a Ph.D. degree in Physics at Cornell University NY. Most of the time, however, he was doing experiments in the physiological domain. The physiological direction became definitive after his promotion to Assistant Professor of Physiology at Columbia College of Physicians and Surgeons. In previous experiments on various cells Cole had obtained experience in impedance measurements. It seemed more than logical that with the giant axon he also started in this direction. The giant axon was mounted in a Wheatstone bridge configuration. Upon stimulation and production of an action potential the bridge went out of balance. The unbalance indicated a major increase in conductance, but no change in capacity (Cole and Curtis 1939)(Fig. 5B). Right at the time of performing these critical experiments Hodgkin finishing his 1 year stay in the United States was visiting Woods Hole to say good‐bye. Cole reports: “Hodgkin visited when we had the impedance change on the scope. He was as excited as I've ever seen him, jumping up and down as we explained it”. Everyone will agree that indeed the picture has inherent beauty. These were also the feelings of the Biophysics group who used it as their logo. The picture appeared in the Danish “La Femme” and was used in a newlywed's apartment as decoration. In the meantime the time axis was switched to the impedance change. You will agree there is music in this picture.

The year in New York was very important for Alan Hodgkin on a personal basis. It was there that he met Prof Peyton Rous, Nobel prize, and his daughter Marion who later became Mrs Hodgkin. We will meet Mrs Hodgkin again in this story.

After his return to Great Britain from the stay in the United States, Alan Hodgkin, the Research Fellow at Trinity College Cambridge, asked Physiology Undergraduate Andrew Huxley (Fig. 7) to join him during the summer 1939 in Plymouth for research on the giant axon. It was the start of an excellent fruitful collaboration. Andrew Huxley was a member of the prominent Huxley family. He was the grandson of Thomas Huxley, known as “Darwin's bulldog, the defender of Darwin against the Church of England bishops.” Andrew had two half‐brothers Aldous Huxley (writer) and Julian Huxley (biologist). Andrew was strong in mathematics and skillful in technology. Already at the age of 12 he was given a turner's lathe by his parents. It is not abnormal then that later most of the equipment used in experiments was designed and even built by Andrew. A fantastic example is later the development of the Interference microscope.

In the summer of 1939 the plan of Hodgkin and Huxley was to insert an electrode in the axon and measure the transmembrane potential. They quickly learned that scraping the cell wall with the electrode resulted in a leaky membrane. Andrew Huxley, however, was an excellent technician and was able to mount a combination of two mirrors in such a way that by looking through the microscope one could see in one field the two sides of the membrane facing the electrode.

**Figure 6:**
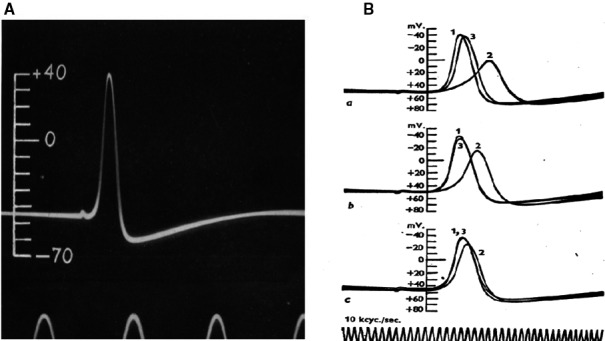
(A) August 1939, edge of the second world war. Measurement of resting and action potential in the squid giant axon with an intracellular electrode. Demonstration of a large overshoot (Hodgkin and Huxley 1939). (B) Postwar 1947 after the damage reconstruction of the Plymouth laboratory. Confirmation of the sodium hypothesis: the action potential amplitude of the squid giant axon changes as expected for a sodium electrode (Hodgkin and Katz 1949). Reproduced with permission.

After solving this practical problem the outcome of the experiment was clear. The resting potential was in the order of −50 mV and upon stimulation the potential went not only to zero but reversed. There was thus a large overshoot (Fig. 6A). They had no time to confirm the results by performing more experiments because the war was imminent. A short letter was sent to Nature, one printed page, and contained only a summary description of the results, no discussion. At the same time (1939) Cole and Curtis were also trying to insert an electrode and they too were successful. They used, however, an AC‐coupled amplifier and thus could not faithfully record the resting potential, a DC signal. It was August 1939 and on September 1 Hitler marched into Poland.

### The postwar period

During the war, on both sides of the ocean scientists were enrolled in the army. In the U.S. Cole and Curtis were involved in the Manhattan project and were performing experiments on biological effects of radiation. In Great Britain a similar situation: Hodgkin in the airborn radar and Huxley in naval gunnery. Their time was not completely lost. Both got ample experience in negative feedback mechanisms.

After the war, back to electrophysiology. Having confirmed that there was an overshoot, the next logical step was to test the possibility of an increase in sodium conductance. Hodgkin, however, had serious problems with the sodium hypothesis. It was difficult to see for him how a hydrated Na^+^ ion with a larger dimension than the hydrated K^+^ ion could become more permeant. It was the renewed contact with the outside world that would change the ideas of Hodgkin. Indeed London 1945 was again attracting people from outside. On one of the seminars given by Krogh, a Danish scientist, well‐known afterward for his analysis of capillary circulation in skeletal muscle, Andrew Huxley learned that experiments with radioactive ^24^Na (a byproduct of the war) had shown a high permeability to Na^+^ ions in different animal tissues. During a search in the library, Andrew made another fantastic discovery. In 1902 (actually not such recent reference) Ernest Overton (we mentioned his name already in relation to the permeability of membranes to lipid soluble substances) had published a paper on the indispensability of Na^+^ (Li^+^) ions for the excitability of skeletal muscle (Overton 1902). These two experimental arguments convinced Alan Hodgkin that a test of the Na^+^ hypothesis was imperative. It was 1945 and Plymouth had been heavily bombed and damaged. It took practically 2 years before the experimental work on squids could be restarted.

**Figure 7:**
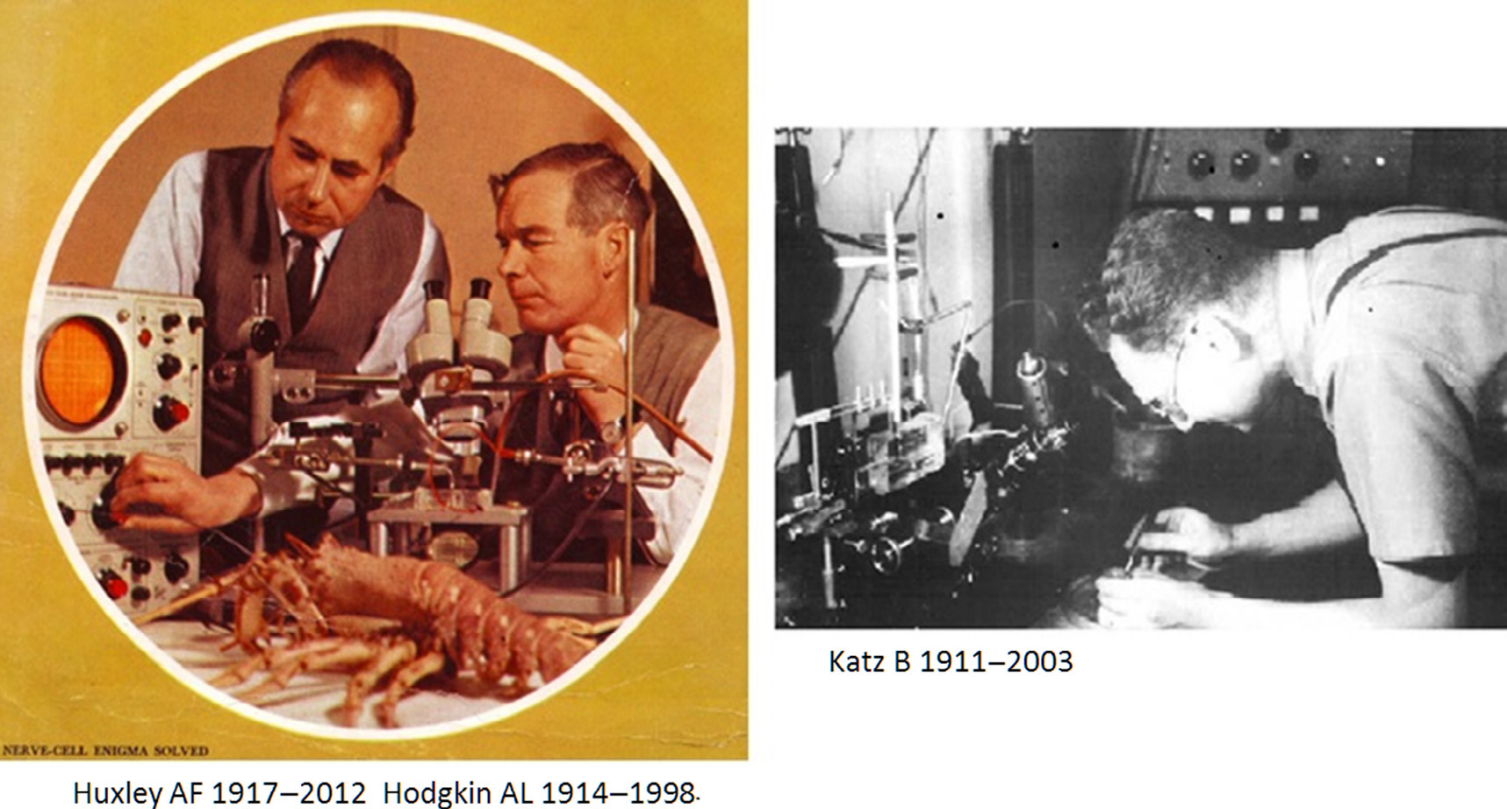
Left: Authors of the voltage clamp saga, winners of the Nobel Prize at work. The cover of the 1963 Nobel Prize Programme, made available by Deborah Hodgkin to Schwiening. With permission. Right: Photograph of Katz B. by Weidmann Silvio. Courtesy Weidmann.

In the meantime Bernard Katz (Fig. 7), back from Australia where he had worked with John Eccles, who won the 1963 Nobel prize in Physiology‐Medicine, joined Hodgkin and Huxley. Bernard Katz from Jewish origin, had left Germany in the mid‐thirties after finishing medicine in Leipzig. He had been involved in muscle research with A.V. Hill University College London and in neurophysiology with J.C. Eccles in Australia. After his war‐stay in the Australian army (he had obtained the citizenship) he returned to University College London and in the summer months he participated in the research of the Cambridge group in Plymouth.

The results in that summer of 1947 were straightforward. The sodium spike was confirmed. In the publication (Hodgkin and Katz 1949) (Fig. 6B) that came out with a substantial delay, the name of Huxley is missing. What happened? Andrew was absent: reason? Away on honeymoon, marriage with Jocelyn Pease, daughter of the geneticist Michael Pease.

The time was ready for a new approach: the voltage clamp. In the late forties both the Cole and Hodgkin teams had been thinking about a novel approach, which consisted of controlling voltage or current over a large surface of membrane. This is evident from an exchange of letters (late 1947). A.L. Hodgkin to K.S. Cole:

“…I am interested in the possibility of stimulating the axon with a diffuse electrode in such a way that the axon is excited uniformly over a length of 1 to 2 centimeters….”

And Cole to Hodgkin: “I am sure that you will be excited to hear that we spent the whole summer with an internal electrode 15 millimeters long and about 100 microns in diameter…”

**Figure 8:**
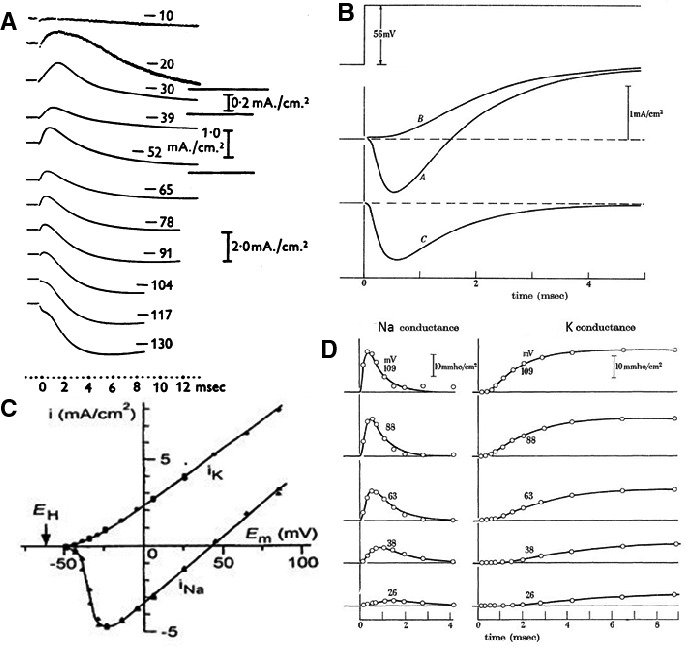
Ionic theory. (A) Compilation of the essential steps from measurement of currents at different voltages, inward current shown as upward deflection; (B) dissection in Na+ and K+ current, (C) current–voltage relation of the two currents and (D) time evolution of conductance at different voltages, resulting in the formulation of equations and calculation of the action potential (Hodgkin 1958). With permission.

Clamp experiments thus were already running in the summer of 1948. In Plymouth the voltage clamp full attack was delayed because of the destructions and came in August 1949. The squid were in good supply and in 1 month of experimentation virtually all the voltage clamp recordings were obtained that were used in the five papers 1952 (Hodgkin 1958). Figure 8 summarizes the essential results. An example of scientific logic: simple, straightforward, well planned: Currents for different voltage steps; followed by a dissection in Na^+^ and K^+^ current, IV relation of the two currents and time evolution of conductance at different voltages.

**Figure 9:**
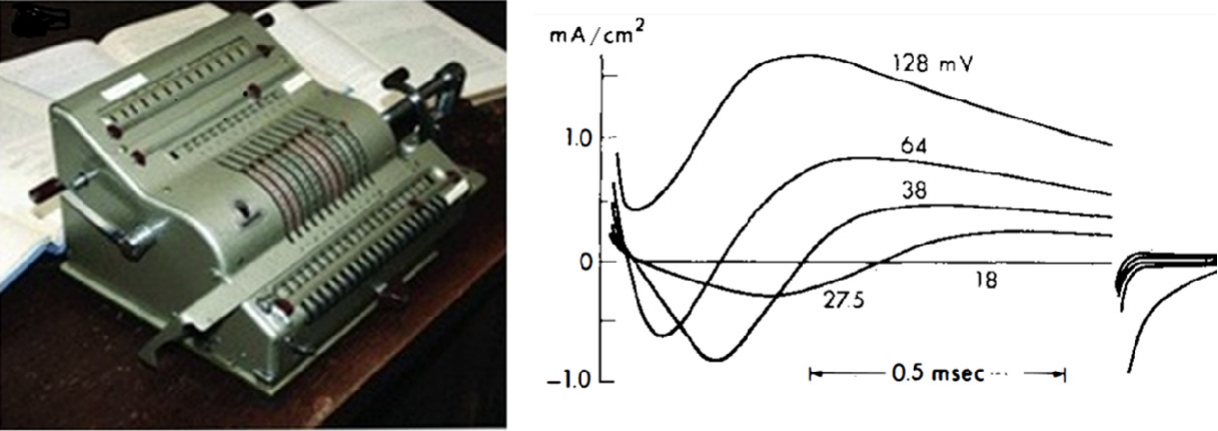
Left: The historical hand‐operated machine, also called “Brains of steel” by the advertisers, which replaced the Cambridge University Computer out of order for 6 months. Photograph taken by Schwiening 2012. With permission. Right: Original voltage clamp currents, recorded by Cole in 1947, published in 1949 (Cole, 1949). Reproduced from (Cole 1979), with permission. Compare with currents of the Cambridge group in Figure 8.

Analyzing and writing followed the experiments. Important questions were: carrier system or voltage‐dependent gating? Hodgkin and Huxley went finally for the system with electrically charged particles in the ionic pathway, the m³h and n^4^ formulation. All equations and constants were ready in March 1951. Finally the action potential had to be computed from the equations. They had hoped to get the help of the Cambridge University Computer but were informed that it was out of order for 6 months. Andrew Huxley decided then to do the calculation using a hand‐operated Brunsviga machine (Fig. 9A). With this “Brains of steel”, and after thousands of rotations of the mechanical calculator crank, he cleared the job in 3 weeks.

The results were satisfying and certainly justified the distribution of the Nobel Prize in 1963. In this context you will forgive me to become a little more personal. In 1958, 5 years prior to the Nobel Prize, Hodgkin was given a doctorate honoris causa at Leuven University on the initiative of the department of Physiology. At that time I was assistant in Physiology and got promoted to act as chauffeur and tourist guide for Professor Hodgkin. As recompense for the guiding I was invited together with my wife to stay for a week with the Hodgkins in their Cambridge House. This was a fantastic experience. I remember very well Mrs Hodgkin going every evening upstairs with her children to read from a book before sleeping. Mrs Hodgkin, American from origin, was editor of the Childrens Book section of MacMillan Publishing Company. And Alan Hodgkin in the morning before leaving for school, testing the memory of his children for citing the Bible's verses by heart. And the Trinity College dinner…no comment.

What happened in the meantime with Cole's group? This is, in my opinion, a sad story. Cole and Marmont, (Curtis left during the war, in 1942), shared a lab at the Marine Biological Station at Woods Hole, but their relationship was far from optimal. Cole preferred the approach of voltage clamp to constant current, Marmont the opposite. They compromised: Marmont used the equipment during the day in the current clamp mode and Cole worked in the evening and the night after switching the installation to voltage clamp by a second plug‐in. At the insistence of Cole they performed a few voltage clamp experiments and were quite successful. This is evident from a comparison of currents recorded by (Cole 1949) (Fig. 9B) and recordings from Hodgkin et al. (1952) (Fig. 8A).

In this respect I may cite some remarks that I found in the memoir on Cole by Andrew Huxley and that illustrate the intellectual and scientific honesty of the Cambridge authors : “These records showed qualitatively all the main features that Hodgkin and I found in our experiments in 1948 and 1949: they showed an appreciable lag between the step of membrane potential and the rise of the transient inward current (due to sodium entry) …Later we fully confirmed the genuine feature of the membrane response, and it was an important factor in determining the formulation that we finally adopted in our mathematical representation of the permeability changes (Hodgkin 1958)”. Cole had, however, problems with the interpretation of his findings. Marmont apparently performed many experiments but even did not try a quantitative description and left the field (Marmont 1949).

### The Intracellular Microelectrode 1949

In the rest of the review I will concentrate on information related to cardiac tissue and cells. This section does not include spontaneous activity and propagation, topics which will be treated separately.

#### Gerard, Graham, Ling: the beginnings

**Figure 10:**
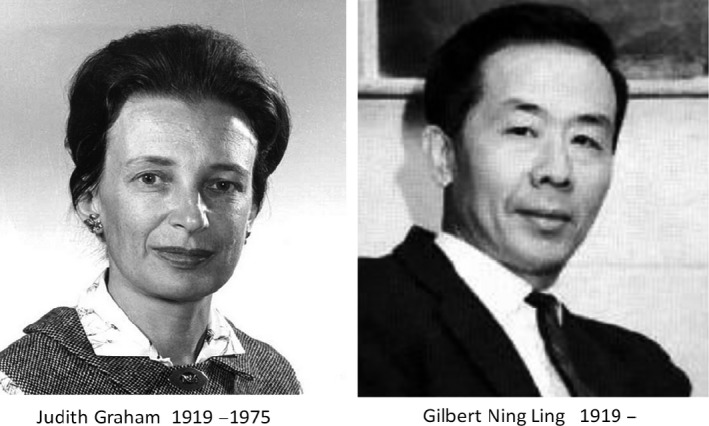
Two Ph.D. students at the Chicago University Judith Graham (1946) and Gilbert Ning Ling (1949) were at the start of the microelectrode saga. Their director at the Department of Physiology was Ralph W. Gerard. The photograph of Graham was granted by the Stanford Medical History Center; photograph of Ling by Wikipedia.

Around the time the first voltage clamp experiments had resulted in the formulation of “The ionic theory,” another important technical innovation happened, the birth of the intracellular microelectrode. It created a major step forwards in electrophysiology. It took place in the department of Physiology University of Chicago with Professor Ralph W. Gerard (1900–1974) as the head and Ph.D. students Judith Graham and Ning Ling in succession at the bench (Fig. 10). The aim was to fabricate a micropipette that could be inserted in excitable tissues and record transmembrane potentials.

A first attempt was made by Judith Graham in 1946, but results were not optimal (Graham and Gerard 1946). The diameter of the electrode tip was above 1 *μ* and caused too much lesion. The start was given, however, and Judith Graham obtained her well‐deserved Ph.D. degree at Chicago University. Later she shifted to clinical research at the Stanford Research Institute, California. She became famous by discovering a method to concentrate the antihemophilic factor or factor VIII in blood plasma. This method shortened and improved substantially the treatment of hemophilic patients. She became a member of the scientific advisory committees of the National Institutes of Health and the National Hemophilia Foundation, which renamed its research fellowship the Judith Graham Pool Research Fellowship in her honor.

The second attempt in the microelectrode development was reserved to a Chinese student in biology, Gilbert Ning Ling. In 1944 Ling won a scholarship to continue his studies of biology in the United States. In Chicago his assignment was to improve the quality of the microelectrode developed by Judith Graham. He fulfilled this requirement by reducing the tip diameter to 0.5 *μ* or less and correcting the tapering. The publication followed in 1949 and became the text that most of the young researchers of my generation had to read and eventually translate in practice by fabricating such electrode (Ling and Gerard 1949a; Ling and Gerard b; Ling and Woodbury 1949; Ling and Gerard 1950).

Before Ling's publication the message already was spread by word. In 1948 Alan Hodgkin visited Chicago to meet Cole and discuss plans on voltage clamp of the squid giant axon. On that occasion he also visited Gerard and Ling and learned about making microelectrodes. Hodgkin from his side had his contribution and proposed to use 3 molar KCl solution instead of the isotonic solution in order to lower the electrode resistance.

Ling's early publications were in the style of “the ionic theory.” But already shortly after his first four papers on the membrane potential of muscle cells of the frog, he expressed doubts about the existence of a membrane barrier around cells and the existence of an active transport for Na^+^ ions and channels. He published five books (among them “Association induction hypothesis” and “Physical basis of life” in 1982) and 200 papers trying to disprove the generally accepted view of the cell. In 1988 his laboratory was shut down: stop of the NIH funds. He carried further on with private subsidies. In a publication of 2008 his conclusion was still that the active Na^+^, K^+^ pump does not exist but that the nanoprotoplasm is the ultimate basis of life.

The first electrodes were made by hand after heating with a micro‐burner Bunsen apparatus. The production of microelectrodes was a question of patience and time. Otto Hutter expressed the atmosphere when he started with Stephen Kuffler in Baltimore in these simple, nude words: “I received a room, a bucket, glass and a Bunsen burner.” Later, “puller**”** systems were developed. The by‐hand procedure was not supported with enthusiasm by the scientific community. The story goes that J.C. Eccles, the Nobel Prize winner, had never made a glass microelectrode. A graduate student from Lebanon at the University of Washington, Seattle, Suhayl Jabbur, used this fact as an argument for not following the advice of his tutor to make his own microelectrodes. If Eccles could win the Nobel prize without learning to make microelectrodes, Jabbur argued, then I, coming from a prominent Lebanese family, am convinced that it is a skill that I can do without. According to Halliwell and Whitaker (1987) the main reason for the absence of enthusiasm for the by‐hand procedure was the lack of reproducibility. This is certainly correct but those who have used the method claim that there were also positive aspects in using your hands. They mention for instance that at the moment of withdrawing from the flame of the Bunsen burner they could feel how large the electrical resistance of the electrode would be, naturally with an appropriate standard deviation. On the other hand it is recomforting that at the end of their recommendation Halliwell and Whitaker added: *“*Great artists can heat a capillary in a Bunsen and pull out a fine tip.*”* Many electrophysiologists were great artists.

#### Application of the microelectrode to cardiac cells

**Figure 11:**
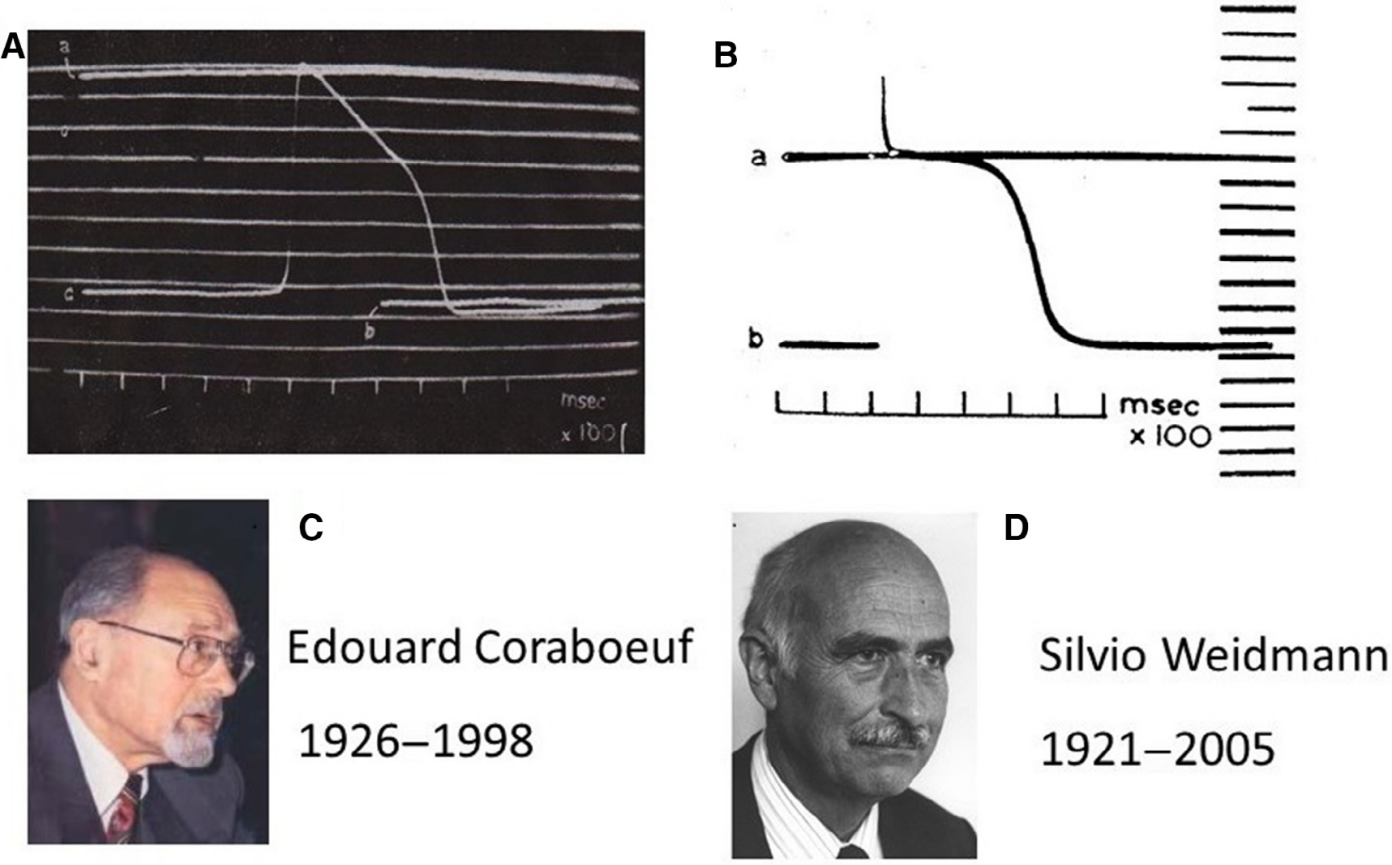
(A) The microelectrode message spread over the world. In Europe Weidmann and Coraboeuf learned the technique during their stay in Cambridge, via A. Hodgkin. Their first recordings were probably too hasty and did not show an overshoot (Coraboeuf and Weidmann 1949a,b). (B) The wrong message was corrected 2 weeks later, showing a strong and beautiful overshoot (Coraboeuf and Weidmann 1949a,b). Reproduced with permission. (C) Photograph of Coraboeuf reproduced with permission from (Escande 1999); (D) photograph of Weidmann, courtesy of Ruth Weidmann.

The first in Europe to use microelectrodes on cardiac cells were Weidmann (1921–2005) and Coraboeuf (1926–1998) (Fig. 11) during their stay in Cambridge with Hodgkin. Their initial result is shown in two communications that were sent to the Comptes Rendus Société de Biologie Paris (Coraboeuf and Weidmann 1949a,b). The Comptes Rendus at that time was the easiest way to a fast publication, because the Société met every fortnight in Paris, also in French provincial towns and in other countries, such as Switzerland and Belgium, where people were supposed to understand French. Printing followed in a regular way.

As can be seen in Figure 11A and contrary to expectations from the findings in the giant axon the upstroke of the action potential barely reached the zero level. Was the Purkinje fiber not following the rules of the giant axon and showing a nonselective increase in permeability as predicted by Bernstein? The answer was given in a communication 2 weeks later in the same Comptes Rendus. A marvelous overshoot (Fig. 11B) was seen and Silvio explains:

“The action potentials showing no overshoot were taken on August 18th, shortly before Edouard Coraboeuf left from Cambridge. Also, the two ap's of the first communication were the only one we had at that time. On Aug 29th I recorded the first overshoot, much to the relief of Alan Hodgkin. We asked Ali Monnier to suppress the first communication. But he had already communicated it to the Soc Biol Biol de Paris and did not feel ill about the story.”

What had happened? We listen again to Weidmann: “We should be aware of the fact that in 1949 thermostats were not available in the cellar lab of Physiology in Cambridge. Perfusion solutions had therefore to be warmed up by Bunsen burner. Because the distance between the perfusion bath and the warming site was rather long and the temperature in the room not very high the temperature in the flask that was warmed up had to be risen to levels where the Ca^2+^ salts went out of solution and precipitated.” The Purkinje preparations went thus in a nonphysiologic situation of Ca^2+^ deficiency and our two electrophysiologists discovered unexpectedly that Ca^2+^ ions have a strong effect on the rise in Na^+^ conductance.

Via Alan Hodgkin, Weidmann and Coraboeuf were responsible for the spread of the microelectrode in Europe. When Weidmann showed Alan Hodgkin the first transmembrane action potentials with a nice overshoot, Hodgkin said to Silvio: You can now reinvent and translate in modern terms the most important aspects of cardiac electrophysiology. And yes, that happened.

On the U.S. east coast, Brian Hoffman (1925–2013) in Chandler McC Brooks’ lab, State University New York, learned the microelectrode technique by reading Ling's original paper. Brian had studied medicine at the Long Island College of Medicine and was doing an internship in New York when illness forced him to interrupt his clinical training. He applied at the Downstate Medical School and was accepted by Professor Brooks in the Department of Physiology. It was 1949, the year of Ling's publication on the microelectrode. Brian Hoffmann was so enthusiastic about the electrophysiologic research using a new technique, that his temporary interruption of clinical training became a definitive shift. His first aim was to understand the electrocardiographic records obtained from extracellular electrodes in terms of changes in transmembrane potentials at the cellular level. He was joined by Paul F. Cranefield, Ph.D. (1925–2003). The union of these two exceptional, supplementary personalities was very fruitful. It resulted in a clearly written Physiological review paper in 1958 (Cranefield and Hoffman 1958), followed by a remarkable monograph in 1960, Electrophysiology of the Heart (Hoffman and Cranefield 1960). The book, according to Weidmann represents “a comprehensive text, readable by basic scientists as well as by clinicians (that) has been essential in narrowing the gap between the disciplines.” A true example of efficient translational approach. The publication was a success, and of such a scale that a pirated Russian translation appeared in 1962. An authorized Japanese translation was published in 1977. In 1981 it was added to the list of “This week's citation classics” of Current Contents. An important objective in the Department of Physiology next to productive research was education. This was already the case before the microelectrode advent but was now amplified; the center became a mecca for established researchers and young students interested in obtaining a PhD degree. Brian enjoyed to act as mentor and teacher. From the American Heart Association he received the Academic Mentorship Award. In 1963 Hoffman and Cranefield moved to Columbia University College of Physicians and Surgeons, Department of Pharmacology. A few years later P Cranefield shifted to Rockefeller University. He was asked to become the Editor‐in‐Chief of The Journal of General Physiology and remained in function for almost 30 years. Brian Hoffman continued at Columbia University. He remained so involved in academic work that his retirement was three times delayed. Because the announcement of the delay was rather late the planned festivities went on. A first time in 1990, with a fantastic retirement symposium in Islamorada in Florida, a second time 5 years later and again shortly before the fatal date Brian changed his mind. On the second delay there was a restricted dinner for the former Downstate and Columbia colleagues in the Hudson River Club. And for the third time restriction resulted in a dinner for two.

Walter Woodbury was responsible for the spread of the microelectrode technique to the West coast of the United States and with his brother Lowell to Japan. Walter Woodbury, Ph.D. student of Utah University had stayed for 6 weeks with Ling in partial fulfillment of his thesis and learned the technique in detail. His first publication (Woodbury et al. 1950) was on frog ventricular tissue with coauthors Lowell Woodbury, the older brother of Walter, and Hans Hecht, who according to Silvio Weidmann was the first and probably the only chairman Internal Medicine who could pull microelectrodes by hand (another artist) and insert them in frog ventricular preparations (one of the most difficult preparations to use). Woodbury's contribution to the promotion of the microelectrode was substantial. He made numerous studies on different preparations and species, even in the cat life spinal cord and in the human left ventricle during open heart surgery. The spinal cord preparation was further exploited by J.C. Eccles with as result part of the Nobel prize together with Hodgkin and Huxley. Woodbury's group was also the first to derive transmembrane action potentials from the human heart. The experiment was live transmitted via the local Seattle TV. A flexible, floating type of microelectrode, developed by Woodbury and Brady (1956) was used to avoid damage by movement of the muscle. The initial idea of recording from the human ventricle was generated by J. Lee, a student in medicine, who spent his summer in the lab of Woodbury. Actually he was also the performer of the stunt.

#### *“*The reinvention of cardiac electrophysiology*” (dixit Hodgkin)*

**Figure 12:**
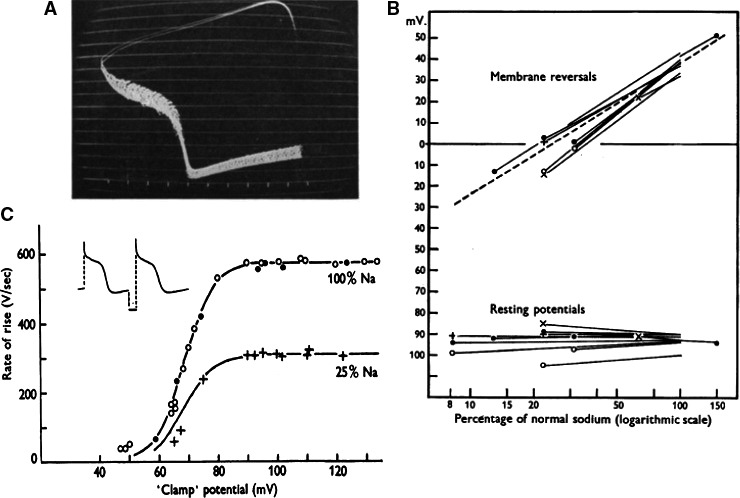
(A) Impedance changes during the course of the Purkinje action potential. Injection of short hyperpolarizing pulses. Voltage calibration in steps of 10 mV. Duration of the flyback of sweep cycle approximately 12 msec (Weidmann 1951a). (B) Membrane reversal and resting potential as a function of percentage normal sodium (Draper and Weidmann 1951).(C) Relationship between “clamp” potential and maximal rate of rise of action potential. Open circles: Tyrode solution, crosses: 25% normal sodium, full circles after changing back to normal.(Weidmann 1955a). With permission.

The upstroke of the cardiac action potential. After having confirmed that cardiac cells show an overshoot the next step was to test whether the sodium hypothesis could also be applied to cardiac tissue. The answer to this question was given in three steps: (1) the membrane resistance during the upstroke of the action potential, as estimated from the shift in potential during the flyback of the cathode ray sweep was found to be dramatically decreased (Fig. 12A) (Weidmann 1951a); (2): the amplitude of the overshoot varied as a function of log external Na^+^ concentration in a way expected for a sodium electrode, whereas the resting membrane potential was unaffected (Fig. 12B) (Draper and Weidmann 1951); (3) the maximum rate of depolarization during the upstroke of the action potential changed as a function of the preexistent resting potential in an sigmoid way, similar to the behavior of the h‐parameter for the squid giant axon (Fig. 12C) (Weidmann 1955a).

**Figure 13:**
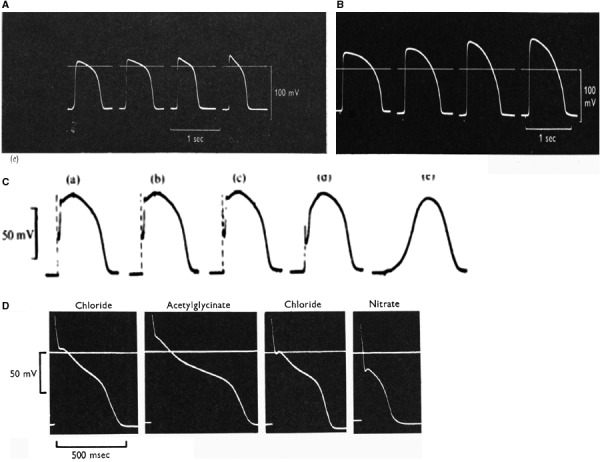
(A) Dependence of action potential amplitude on external sodium concentration in frog ventricle. From right to left: 100, 75, 50, and 25% of normal. The slope of the relationship between overshoot and change in external sodium was only 17.3 mV for a tenfold change (Niedergerke and Orkand 1966a). (B) Effect of change in external calcium concentration: from left to right, 0.3, 1, 3, and 5 mmol/L Ca2 + ‐Ringer (Niedergerke and Orkand 1966b). (C) Action potential recorded in calf Purkinje fibers, presence of adrenaline (5.5 × 10^−6^ mol/L). The sodium current was progressively blocked with increasing doses of TTX. (a): control, (b): 3 × 10^−8^, (c): 3 × 10^−7^, (d) and (e): 3 × 10^−6^ mol/L. (Carmeliet and Vereecke 1969). (D): Action potentials in chloride Tyrode are compared with action potentials in acetylglycinate and nitrate solutions (Carmeliet 1961a). With permission.

Soon, however, deviations from this classic view appeared in the literature (Coraboeuf and Otsuka 1956; Délèze 1959). In the guinea‐pig and the frog ventricle (Niedergerke and Orkand 1966b) the total amplitude of the action potential was found to be less reduced with a fall of external Na^+^ concentration than expected (Fig. 13A) but, instead was more sensitive to changes in external Ca^2+^ (Fig. 13B). In fact the amplitude followed the rule for a Ca^2+^ electrode. On the other hand the maximum rate of depolarization, which occurred earlier during the upstroke, was very sensitive to external Na^+^ and was tending to zero in the absence of Na^+^ in the solution. The results suggest that the upstroke in these preparations consists of two components. An early fast component sensitive to external Na^+^ and obeying the rule for a Na^+^ electrode followed by a smaller component sensitive to external Ca^2+^and approaching the behavior of a Ca^2+^ electrode.

A few years later evidence was presented that the fast Na^+^‐sensitive upstroke and the Ca^2+^‐sensitive slow upstroke could occur independently (Fig. 13C) (Carmeliet and Vereecke 1969). By blocking the rise in Na^+^ conductance via an increase in external K^+^ or addition of TTX and simultaneously increasing the Ca^2+^ conductance by adrenaline the first rapid depolarization due to influx of Na^+^ ions disappears but the secondary depolarization which supposedly is due to an increase in Ca^2+^ conductance remains and is magnified. This results in slowly conducted Ca^2+^ ‐dependent action potentials (Carmeliet and Vereecke 1969). Similar slow‐rising action potentials can be obtained when Ca^2+^ is replaced by Sr^2+^ ions in the absence of Na^+^ in the solution (Vereecke and Carmeliet 1971). Action potentials generated by Ca^2+^ ions are even the rule in invertebrate tissue (Fatt and Ginsborg 1958).

Chloride and early repolarization (Fig. 13D). In Purkinje fibers substitution of Cl^−^ ions by acetylglycinate has a weak effect on the resting potential but the fast repolarization following the peak of the upstroke is reduced in amplitude and slowed down. When nitrate replaces Cl^−^ ions on the other hand the peak is accentuated and the action potential duration shortened. In measurements of membrane slope resistance at different levels of membrane potential, substitution of Cl^−^ by acetylglycinate or nitrate had an increasing, respectively, decreasing effect on the resistance. These findings suggested that the permeability of Purkinje fibers to Cl^−^ anions is small at the resting potential but increases upon depolarization and may play a role in the rapid repolarization and genesis of the notch to the plateau (Carmeliet 1961a).

Plateau resistance and Inward rectification; fall in K^+^ conductance upon depolarization. In his monograph Weidmann enumerates different mechanisms for the generation of the long plateau but not the possibility of a fall in potassium conductance; although according to a short communication in Amer J Physiol (Weidmann 1955b) he had measured the membrane slope resistance at different potentials and found an increase in resistance by a factor 4 at levels corresponding to the plateau. An apparent memory gap? Weidmann had doubts about the method used, and probably more important he did not see why heart cells should be different from the giant axon: logic demanded that an increase in Na^+^ conductance should be followed by an increase in K^+^ conductance. This is evident from the wording in the short note of the American Journal of Physiology: “The present findings may indicate that the *permeability* of the cardiac fiber membrane to K^+^ ions *does not increase appreciably* under the influence of a longlasting depolarization while the Na^+^ permeability becomes approximately equal to the K^+^ permeability. And in another comment: “Since gK in the membrane of the squid axon rises upon depolarization, it did not occur to me that one might assume gK to fall in cardiac cells.” The situation changed, when one day in 1959 Edouard Coraboeuf came from Paris to Bern with a manuscript in his luggage (Coraboeuf et al. 1958) and tried to convince him of a voltage‐dependent drop in conductance during the plateau. “I felt somewhat heretical toward Hodgkin and Huxley but had to give in.” From that moment Silvio Weidmann was ready to reinterpret his findings published in the 1955 abstract of the Am J Physiol.

**Figure 14:**
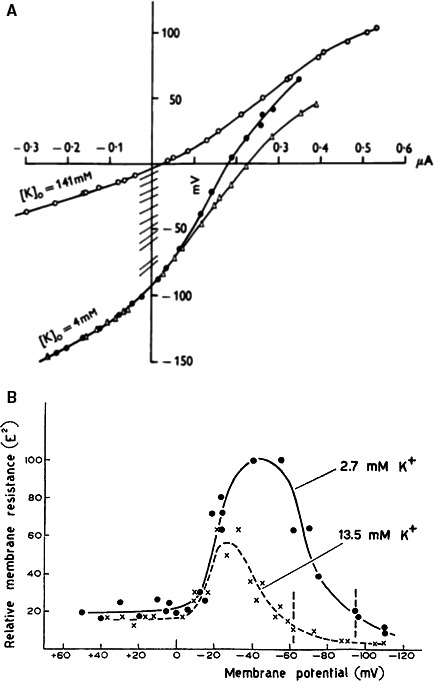
(A) Current–voltage relations in 4 and 141 mM external K^+^ concentration in sheep cardiac Pu fibers. The lines cutting the voltage axis are resistance measurements during the change in potential between the two extremes. Application of long rectangular currents (Hall et al. 1963). (B) Relative membrane slope resistance of sheep cardiac Purkinje fibers as a function of the membrane potential in two external K^+^ concentrations. Short pulses were superimposed on long polarizing pulses to depolarized and hyperpolarized levels (Carmeliet 1961a). With permission.

The first measurements of membrane resistance were made by Weidmann (Weidmann 1951b) in the presence of external sodium ions and did not allow a straightforward explanation in terms of a fall in K^+^ conductance. Measurements of resistance were repeated in Na^+^‐free solutions over a broad range of potentials by the constant current method, independently by Hutter and Noble (1960), Hall et al. (1963) and by myself (Carmeliet 1961a). The method was different in so far that Hall et al. passed long constant currents starting from the resting potential (Fig. 14A) and I used short hyperpolarizing pulses on top of long pulses to different levels (Fig. 14B). Results were quite similar. At normal external K^+^ concentration (2.7–4 mmol/L) the IV relation showed inward rectification; the relative slope resistance increased from 1.0 at the resting potential to a maximum of about 5.0 at −40 mV, to decrease at more depolarized potentials (around the zero level) to 1.0. At higher K^+^ concentrations (13.5 mmol/L) the resistance at the resting potential decreased and the relative increase at depolarized levels was smaller. Above the zero voltage level the resistance was independent of the membrane potential and the external K^+^ concentration.

**Figure 15:**
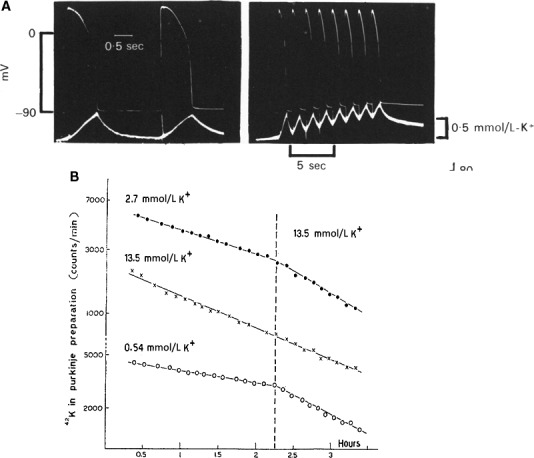
(A) Pulsatile K^+^ concentration changes in the extracellular space measured by K^+^ sensitive electrodes at different rates of stimulation in frog ventricle (Kline and Morad 1976). (B) Effect of external K^+^ concentration on the rate of 42K^+^ efflux in sheep Purkinje fibers (Carmeliet, 1961a). With permission.

Final rapid repolarization. In experiments with radioactive ^42^K^+^ on the turtle ventricle, Wilde et al. (1953) showed that each action potential is associated with a relatively large discharge of K^+^ ions in the vascular bed. The pulsatile K^+^ loss was assumed to result in a temporary elevation of the local K^+^ concentration close to the membrane. Such a rise in local K^+^ was clearly demonstrated in the frog ventricle using K^+^‐sensitive electrodes (Kline and Morad 1976). Did this rise in external K^+^ favor repolarization? To provide an answer to this question, Weidmann (1957) used the slowly contracting turtle ventricle with a plateau of 4 s and equipped with a coronary circulation, the same preparation Wilde and O'Brien had used. He injected a slug of K^+^ rich solution in the coronary artery during the course of the long action potential and observed an early repolarization followed by a transient decrease in diastolic membrane potential. A possible explanation is that an increase in extracellular K^+^ concentration causes a rise in electrical membrane conductance (Fig. 14), due to a larger K^+^ efflux (Fig. 15B) and by way of a positive feedback results in a higher extracellular K^+^ level. In my starting years as a student in medicine I have used the hypothesis of a rise in K^+^ concentration as part of an explanation for the shortening of the frog cardiac action potential with frequency (Carmeliet 1955; Carmeliet and Lacquet 1958) (Fig. 15).

## Voltage Clamp Cardiac Tissue: Multicellular Preparations

### Insufficiencies, Problems, and Critique

Progress in basic research is conditioned by the development of new technical tools. The invention and application of the voltage clamp to the giant axon was certainly such a step forwards. Was this also the case with the application of the voltage clamp to cardiac tissue?

**Figure 16:**
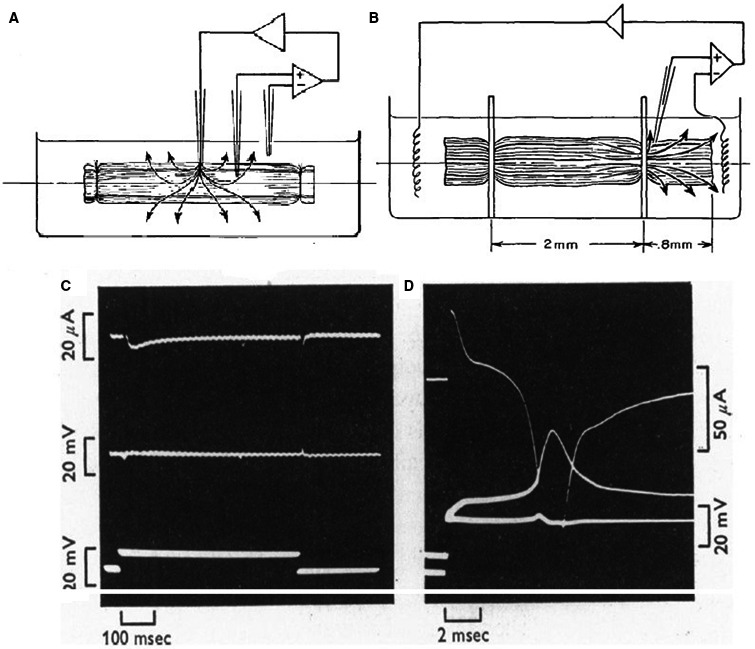
(A) The two‐microelectrode voltage clamp. Current is injected through the electrode in the middle of the preparation; membrane potential is measured between an intra‐ and extracellular electrode (Deck et al. 1964). (B) Sucrose gap method (Rougier et al. 1968). Current is injected in the left compartment, passes through the fibers of the preparation in the sucrose gap, through the membrane of the cells in the right compartment and is measured. Membrane potential is recorded between an intracellular and extracellular electrode. Figure 16A and B are reproduced from Fozzard and Beeler (1975), with permission. (C) Tests of voltage stability in time. Dog ventricular trabecula in sucrose gap, single intracellular microelectrode. Potential difference between two internal microelectrodes (middle trace) during the flow of a small inward current (upper trace), excited by a 10 mV depolarizing current from holding potential of −40 mV to inactivate the INa. (D) potential difference between an intracellular and an extracellular electrode (middle trace) during a large rapid inward current (INa upper trace) showing escape; holding potential −80 mV (Beeler and Reuter 1970b). With permission.

**Figure 17:**
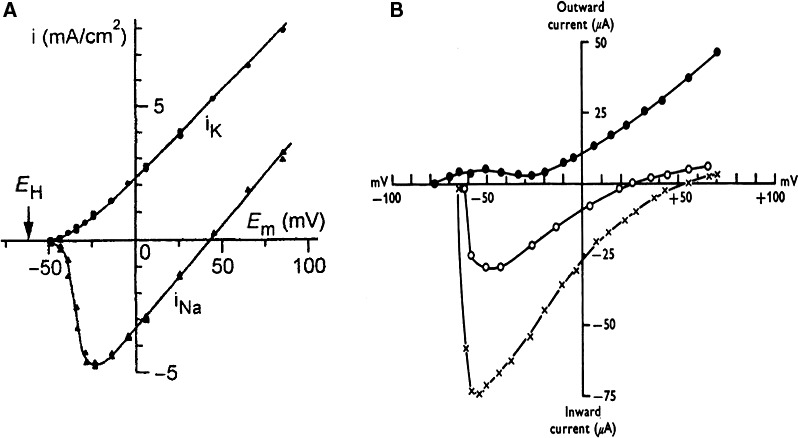
Comparison of voltage clamp results in squid giant axon (long and large intracellular current and voltage electrodes) and in dog ventricle (two microelectrodes). (A) Current–voltage relation of the Na+ and K+ currents in the squid giant axon (Hodgkin et al. 1952). (B) Current–voltage relation of the peak Na+ current in normal Tyode (x) and in solution with 31% of normal Na+ concentration (o). Positive (outward) currents were measured at the end of the 500 msec clamp step. Dog ventricle. Sucrose gap and single intracellular microelectrode (Beeler and Reuter 1970b). With permission.

The method came under two formats: the two‐microelectrode (Deck et al. 1964; Deck and Trautwein 1964) (Fig. 16A) and the sucrose gap voltage clamp (Rougier et al. 1968)(Fig. 16B). For an efficient voltage clamp the membrane potential of the cardiac preparation should be kept uniform in time and in space. A serious problem is the existence of a resistance in series with the membrane. Components of this resistance are: the current carrying microelectrode, the clefts in the extracellular space, the endothelial layer. Especially in Purkinje fibers the contact of the deeper lying cells within the bulk solution is formed by narrow intercellular clefts that may represent an appreciable resistance to current flow. Voltage control is sufficient when the inward current is small (Fig. 16C) but too slow and insufficient with possible escape phenomena as a consequence when the current is high (Fig. 16D). In the cases of instability the current voltage relation is shifted to more negative potential levels with a steep increase in current between threshold and maximum intensity (Fig. 17). The current at these potentials are overestimated and underestimated at more depolarized levels (Beeler and Reuter 1970a,b; Coraboeuf 1978). Existence of a series resistance causes also a prolongation of the time necessary for charging the membrane capacity resulting in serious overlapping with the time course of sodium and/or calcium currents. A further complication of the narrow extracellular spaces is the occurrence of ion depletion and accumulation. The result is misinterpretation of reversal potentials, generation of fake currents, difficult distinction between inward and outward currents (see paper on sinoatrial node pacemaker). In comparison with Purkinje fibers, the clefts in atrial and ventricular muscle preparations are less narrow. However, the total number of cells each surrounded by an extracellular space is not negligible (estimated at 1000 cells in transverse section) and constitutes an important resistance.

Apart from the time problem one should also consider that the size of the preparation to be clamped should be kept small and certainly smaller than the space constant at rest. The effect of increasing the conductance for a certain ion is to reduce the space constant and thus the area which can be voltage‐controlled. In the case of the sodium current which is accompanied by a hundredfold increase in membrane conductance the space constant has been estimated to be reduced to 0.2–0.5 mm (New and Trautwein 1972).

Most of the clamp conditions in early publications did not fulfill the above mentioned requirements. The conclusion is that the sodium current and in some instances even the fast component of the Ca^2+^ current was not clamped efficiently. In “The surprising heart” (Noble 1984) writes (p25): “Thus, it is likely that the first slow inward current recorded by Reuter (1967) in Purkinje fibers contained virtually no iCa.f, since it reached a peak at a time when most of the fast current would be already inactivated.” The overlapping of the long capacity artifact with the early fast current (Na^+^ and Ca^2+^) was probably the reason why the Ca^2+^ current has been described as a slow current, or “courant lent.” The fast part of the current was indeed lost in the capacity artifact and only a slower part of the Ca^2+^current mixed with other currents (NCX, cation current, and Iti) was recorded (see Noble 1984).

Hard judgments have appeared in the literature (Johnson and Lieberman 1971); see the following citation about the use of the shortened Purkinje fiber: “These factors, together with the inordinate length of the preparation of 1–2 mm compared to the resting dc length constant, in our view, puts this combination of voltage‐clamp method and preparation in first place as the most unsanitary experimental setup that has been made available….,” and

“…Furthermore, not only is the complex morphology of the preparation oversimplified, but the nonuniformity of membrane potential control that must occur, at least at some times, in a preparation of such length and with such deep narrow clefts between fibers is completely disregarded” (Johnson and Lieberman 1971).

The critique was justified, not always the wording. In defense of the authors who published disputable results I should mention that in many cases the authors were more or less aware of the shortcomings in which they had to maneuver. The attentive reader will take into account short comments on the side line like the following : “The appendix shows that while these measurements do not *analyze the inward component quantitatively*…” (Beeler and Reuter 1970b). One cannot ignore the existence of a general feeling of uneasiness; fortunately, as a positive reaction there was also the conviction that the development of single cell preparations or a different type of electrode could lead to a solution. Scientists became aware of the necessity to diminish the size of the preparation. See, for example, the use of grids for “crushing” Purkinje fibers and reduce the practical dimension of the Purkinje preparation to 1.0 mm (Aronson et al. 1973). They became also more critical in testing the current passing electrodes for ability to pass large currents and to clean if necessary the tips of the glass microelectrode to get rid of junction potentials. They tried to avoid problems by restricting the experimentation to slow and small currents. Notwithstanding all the critical objections, the information gathered from voltage clamp experiments was not entirely useless. Therefore before entering the era of the patch clamp and the single cell it seems useful to make a short overview of data obtained with the “classic” voltage clamp.

### Overview of data obtained with the early voltage clamp

As explained in preceding pages, a faithful recording of the fast sodium current was impossible (Reuter and Beeler 1969). Even the calcium current was left out. The response times were in the order of a few msec while *μ*sec are required to avoid overlapping of the capacity and ionic currents. Also the size of the preparation was too large with escapes and voltage deviations as large as 50 mV. Some improvement occurred by being more severe in selection of preparation and electrodes and reducing the size of the preparation.

However, although the initial period was difficult because of technical insufficiencies, the period will be remembered as the period when the pacemaker activity was explained by a voltage‐ and time‐dependent pure K^+^ current, the IK2. It was the time that the electrogenicity of two exchangers has been proven. The Na^+^–K^+^ pump is metabolically fueled directly by ATP hydrolysis, the Na^+^–Ca^2+^ exchanger uses the Na^+^‐gradient set up by the Na^+^–K^+^ exchanger.

#### The Na^+^‐K^+^ exchange transport

The Na^+^–K^+^ exchange transport in cells has been shown to be due to an enzyme described by Skou (1957). It was the first enzyme known to produce an electric current with a stoichiometry of 3 Na^+^ ions for 2 K^+^ ions (Haas et al. 1970; Isenberg and Trautwein 1974), pharmacologically blocked by cardiac glycosides.

#### The Na^+^–Ca^2+^ exchanger current (INCX)

A coupling between Na^+^ and Ca^2+^ movement was first suggested as an antagonism of action between both ions on heart muscle contraction (Lüttgau and Niedergerke 1958), and later described more directly as an exchange mechanism (Reuter and Seitz 1968). A more detailed analysis revealed the existence of electrogenicity with a stoichiometry of three sodium ions for one calcium ion (Horackova and Vassort 1979). It plays an important role in regulating intracellular Na^+^ and Ca^2+^ concentration. It acts as an inward current during the plateau and the diastolic pacemaker potential.

#### The positive dynamic current, Iqr

In the same period a description was given of the Iqr, a current that did not change in name but split in two, and changed in mechanism of activation and ionic nature. The first voltage clamp records showed an outward current for depolarizations positive to −20 mV. It was sensitive to Cl^−^ion substitution (Dudel et al. 1967a; Fozzard and Hiraoka 1973) and therefore proposed to be carried by Cl^−^ ions. The following observations, however, caused a shift from a voltage‐activated Cl^−^‐current to a Cai^2+^‐activated K^+^ current: the current was found to be blocked by Mn^2+^ and D600, and injection of EGTA (Siegelbaum et al. 1977); it vanished when Ca^2+^ was replaced by Sr^2+^ or Ba^2+^ (Siegelbaum and Tsien 1980), and by adding TEA or 4‐AP (Kenyon and Gibbons 1979).

**Figure 18:**
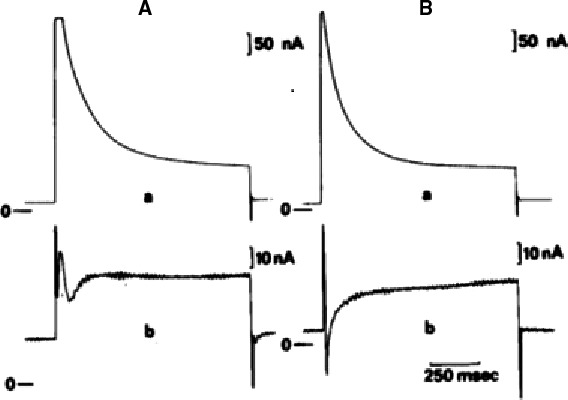
Analysis of the positive dynamic current, Iqr. Effect of 4‐AP and caffeine on transient outward currents elicited in a sheep cardiac Purkinje fiber by depolarizing pulses from a holding potential of −55 mV to −15 mV. (A): Control (a) and 1 mmol/L 4‐AP (b). (B): 10 mmol/L caffeine in the absence (a) and presence (b) 1 mmol/L 4‐AP (b). (Coraboeuf and Carmeliet 1982). Reproduced with permission.

Was the hypothesis of Iqr being a Ca^2+^‐activated K^+^ current final? In 1981 Edouard Coraboeuf decided to make a break and came to Leuven for a sabbatical. We used an improved version of the sheep Purkinje preparation for voltage clamp developed by Aronson in which the size of the short Purkinje segments was reduced to 1.0 mm using a compression wire grip (Aronson et al. 1973). This study (Fig. 18) resulted into the description of Iqr as the sum of two currents (Coraboeuf and Carmeliet 1982);caffeine (Fig. 18Ba and Bb) was used to block a calcium‐activated component, and 4‐AP (Fig. 18AB and Bb) to block a K^+^ current component. The results were as follows: the total transient outward current consists of: (1) a short component which was caffeine sensitive and was assumed to be Ca^2+^‐activated; its ionic nature unknown (see section patch electrode): it was named Ibo for “brief outwards”; presently it is called Ito2. (2) a long component and of greater amplitude, Ilo for long outward, presently Ito1. This latter component showed slow inactivation and was not Ca^2+^‐activated because it resisted treatment with Mn^2+^, caffeine and Sr^2+^ ions. We assumed it being a K^+^ current, because Vereecke et al. (1980) had observed that repetitive activation of this current was accompanied by an increased ^42^K efflux under voltage clamp conditions and 4‐AP suppressed the extra K^+^ efflux as well as the dynamic outward current.

#### Delayed rectifier currents

In Purkinje fibers two delayed K^+^ currents have been described (Noble and Tsien 1969). The first Ix1 is activated between −50 and +10 mV with time constants of 0.5 to 0.7 sec and the second, Ix2 is activated between −40 and +20 mV with time constants in the order of seconds. The x stands for “undecided ion nature.” The reversal potentials indeed were around −80 mV for Ix1 and ‐30 mV for Ix2. The Ix2 is probably due in part to K^+^ accumulation. In the frog atrial muscle two outward currents Ixfast and Ixslow have been described. For very long depolarizations a third component I3 has been found and considered to be due to K^+^ accumulation (Noble 1976).

In mammalian muscle an increasing outward current upon depolarization was absent or small in sheep, calf, dog, but a regular finding in cat and guinea‐pig. In the cat the current has the characteristic of a pure K^+^ current, IK (McDonald and Trautwein 1978). For very long depolarizing clamps a very slow (time constants of many seconds) outward current is found but probably due to K^+^ accumulation. Later IK has been dissected in two components, IKr and IKs (Sanguinetti and Jurkiewicz 1990).

## Single cells, the Suction Electrode, and the Patch Electrode

**Figure 19:**
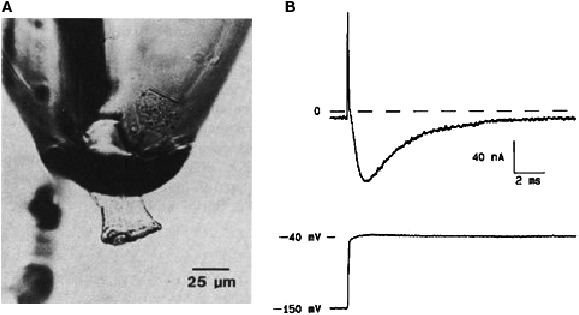
(A) Photograph of a single canine Purkinje cell in the suction‐perfusion pipette. (B): Current response to a voltage clamp depolarization from −150 mV to −40 mV imposed through the suction pipette (Makielski et al. 1987). With permission.

**Figure 20:**
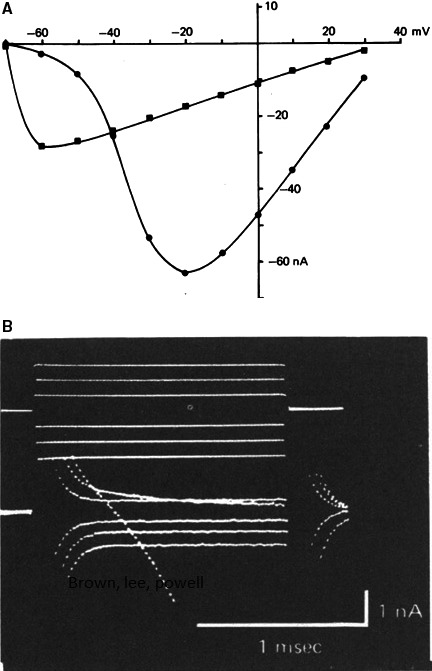
(A) Current–voltage relationships of sodium current obtained using single suction pipette (left) and double suction pipette (right). Holding potential was −80 mV. Same cell in both cases (Brown et al. 1981b). (B) Current traces illustrate separation between capacitive current transients and activation of the Na+ inward current. Voltage and current records using two suction pipettes for voltage clamp of a rat ventricular cell. Notice minimal overlapping of capacitive and current signal (Brown et al. 1981b). With permission.

In the late 1970s the image of electrophysiology changed completely with the development of the single cell, the suction electrode and the patch electrode. For the electrophysiologist using the suction electrode the aim was to measure ionic currents without the shortcomings and errors involved in using the microelectrode or sucrose gap voltage clamp. Patch electrophysiologists wanted to measure ionic currents through single channels. It is the irony of historical development that, at the end, the patch group provided unexpectedly (Neher in his Nobel prize lecture called it “Unexpected benefits”) also the solution to the first group. Indeed the whole‐cell formula is the method of excellence to obtain information on (cardiac) ionic currents at the cellular level. This does not mean that the suction electrode was without success. On the contrary, excellent data on sodium current in cardiac cells have been obtained with the suction electrode(s). As examples I may refer to experiments on single Purkinje cells by Makielski et al. (1987) using a suction‐perfusion pipette (Fig. 19) and Brown et al. (1981a) using the two suction pipette method on rat single cardiac cells (Fig. 20). In this last case capacitive and sodium current signal were nicely separated but this result required the application of two suction pipettes. See the inadequate clamp when using a single suction electrode in the experiment of Figure 20. The advent of the patch electrode also did not exclude the efficient use of the two microelectrodes method on single cells. Compared to the suction electrode, the patch electrode is not only more efficient but also much easier to use; it has moreover a much broader application domain. A progressive shift to the patch type was inevitable (Hamill et al. 1981)

**Figure 21:**
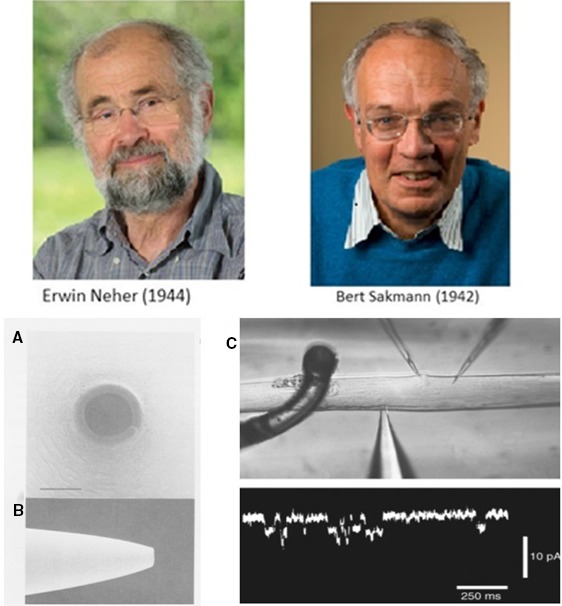
Photograph of Erwin Neher‐1944 and Bert Sakmann‐1942. Permission to use the photographs was granted by the Max Planck Institute of Biophysical Chemistry. The Nobel Prize in Physiology or Medicine 1991 was awarded jointly to Erwin Neher and Bert Sakmann “for their discoveries concerning the function of single ion channels in cells.” (A) Scanning electron micrograph of a tip‐on view of the pipette opening. The darker ring represents the rim of the pipette. Tip opening diameter is 1.1 *μ*m. The width of the rim is 0.2 *μ*m. (B) side‐on view of the same pipette (Sakmann and Neher 1985). (C) Single channel currents from denervated frog (Rana pipiens) cutaneous pectoris muscle. Two microelectrodes to measure membrane potential; below: patch electrode. The patch pipette contained suberyldicholine 0.2 *μ*mole; membrane potential −120 mV (Neher and Sakmann 1976b). With permission.

**Figure 22:**
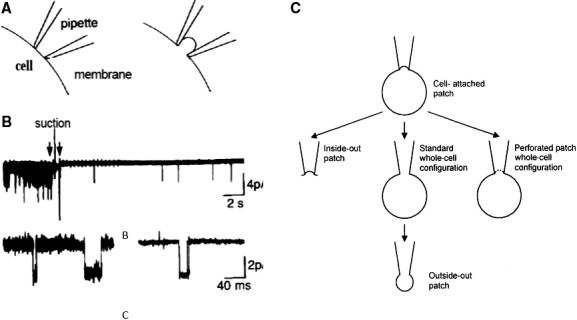
(A) Gigaseal formation between pipette and frog muscle sarcolemma. Schematic diagram showing a pipette pressed against the membrane. The seal resistance is between 50 and 100 MΩ. Applying suction resulted in the formation of a gigaseal; part of the membrane is drawn into the pipette. (B) Current record before, during, and after suction. Suction caused the seal to increase from 150 MΩ to 60 gigaΩ. Notice the fall in noise. Short channel openings are caused by the presence of suberyldicholine. (C) A multitude of possible configurations to measure channels and whole‐cell currents (Hamill et al. 1981). With permission.

The patch electrode has been introduced by Neher and Sakmann (1976a, fig. 21) and Hamill et al. (1981). It is an extracellular electrode with a large mouth diameter of a few micrometers, and thus of relatively low resistance (Fig. 21A and B). The electrode tip is fire‐polished to make a smooth tip and the electrode is filled with a solution approaching either the extracellular or the intracellular ion composition. When the tip is slightly pressed on the membrane surface small suction pulses are applied, the electrode makes a seal to the membrane, providing a good isolation between the inside of the electrode and the outside medium. The resistance of the seal is typically of the order of 10 gigaΩ (this justifies the name gigaseal), ensuring that current leak between the electrode and the extracellular medium is negligible. It takes a few sentences in this text to describe how the gigaseal was born. It took Neher and Sakmann (1976b) about 5 years, between the 1976 first paper on acetylcholine sensitive channels in the neuromuscular junction (Fig. 21C) and the well‐known master paper of 1981 (Hamill et al. 1981), to discover that suction was the secret to prevent noise from the records and enter the era of the gigaseal. Please appreciate the suction effect in Figure 22. Apparently it is not only suction as such which is important; according to an advice of Bert Sakmann it should be preceded by blowing a little. Like so many discoveries in science the mystery unraveling took place on a Saturday afternoon. Erwin Neher did not know what had happened when the signal from very noisy suddenly had changed to a clean recording. The next Monday, however, every collaborator in Göttingen knew, and was using the trick, enjoying research.

Many configurations of the patch electrode have been developed (Fig. 22C): cell‐attached, perforated‐patch, macro‐patch, outside/out, inside/out. The possibility exists to apply intracellular dialysis or to avoid it. For cardiac electrophysiologists it is the ideal method which replaces the former voltage clamp methods and the use of multicellular preparations. The electrode in its whole‐cell configuration has a high seal resistance and a low input resistance allowing to pass large currents without leak. By repeating clamps in the cell‐attached mode and averaging the results, information is obtained similar to the whole‐cell configuration.

**Figure 23:**
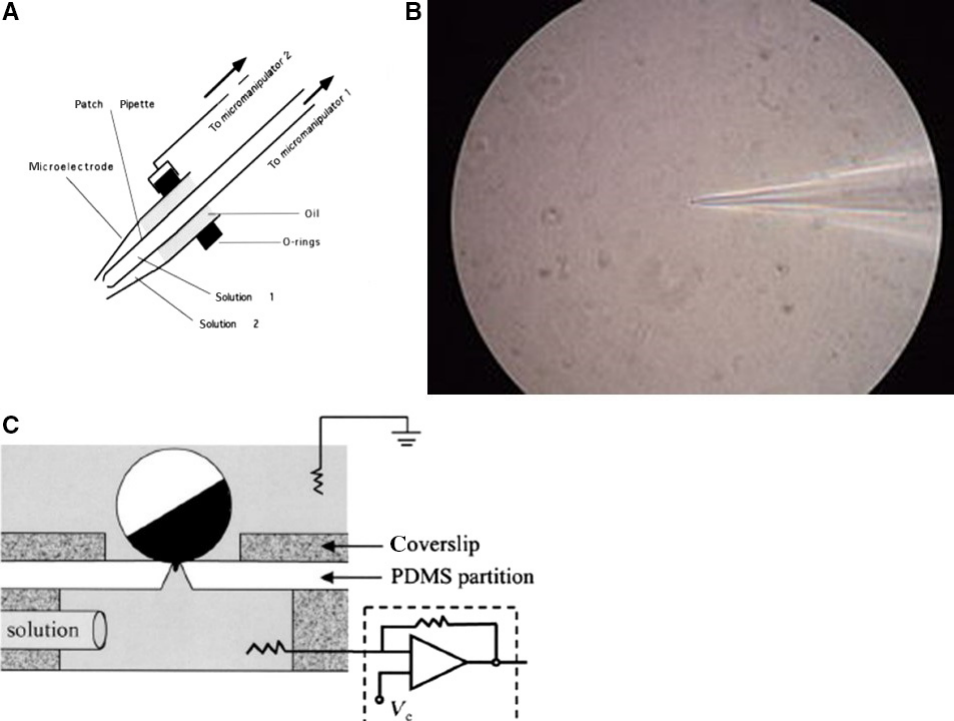
(A) Study of channels in intracellular organelles in vivo. The patch electrode is introduced via a large microelectrode (sharp), which is withdrawn after the patch electrode has entered the cell (Jonas et al. 1997). (B) Study of bacterial ionic channels using the spheroplast. A sheroplast is a structure which is molded from bacterial plasma membranes via culturing in the presence of antibiotics that inhibit cell wall synthesis. (C) On the way to automation. Planar PDMS (polydimethyl siloxane) patch electrode recording of channel currents. Schematic of planar patch recording system. The 200 *μ*m thick, oxidized PDMS partition is sandwiched between bath (upper) and electrode (lower) chambers. A devitellinized oocyte is dropped onto the aperture (8 *μ*m). Gigaseals are possible. Planar system allows for creating arrays of such setup in parallel. High throughput (Klemic et al. 2002). With permission.

The patch clamp can be adapted to measure channels in intracellular organelles (Fig. 23A) such as mitochondrial, nuclear, and endoplasmic reticulum membranes (Sorgato et al. 1987; Mazzanti et al. 1990). Direct application of the patch electrode has been performed on glial cells in culture, on neuron slices in vitro or in brain, on channels expressed in HEK cells. Bacterial membranes can be studied in spheroplasts E Coli (Fig. 23B) which are molded from bacterial plasma membranes via culturing in the presence of antibiotics, that inhibit cell wall synthesis (Kikuchi et al. 2015). The field of interest has been extended to nonexcitable cells, such as secretory epithelia and to the analysis of endo‐ and exocytosis by following changes in membrane capacity.

The versatility of the patch technique is further clearly demonstrated by the fact that it is now used in the industrial search of pharmacologically active drugs. In this application a planar horizontal chip is used to separate two volumes which represent an intracellular and an extracellular medium. The chip is made of sylgart (Klemic et al. 2002) (Fig. 23C), glass (quartz)(Fertig et al. 2002), or polyimide (Stett et al. 2003). Openings of a given diameter in the *μ*m range are made in the chip, cells are added to the upper extracellular compartment and forced to move to an opening in the plastic film by a pressure differential until they form a seal. The forming of seal is still a weak aspect. Only in the case of sylgart, gigaseals of sufficient quality have been obtained (Klemic et al. 2002); in the other cases the seal is still between 1/3 and 1/5 of the desired value and in many other attempts the seal remained in the mega‐Ω range. Using the planar approach up to 384 cells can be studied simultaneously, which facilitates certainly the statistical evaluation of the results (Klemic et al. 2002; Sigworth and Klemic 2002).

### Early advances after the introduction of the single cell and the patch clamp

**Figure 24:**
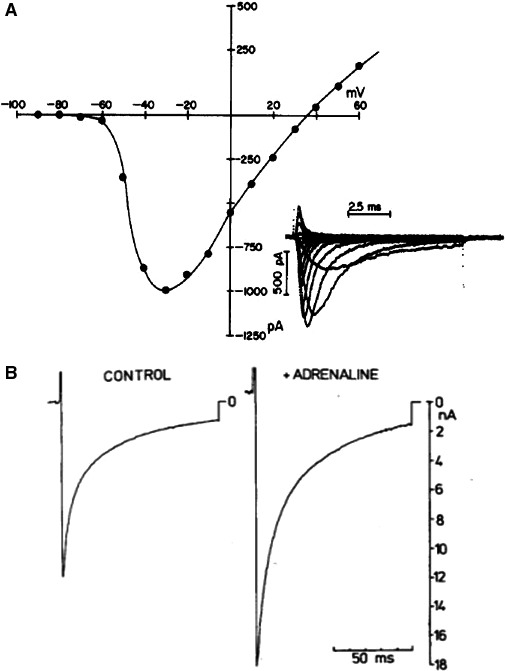
(A) Current–voltage relations for TTX‐sensitive Na+ current from single ventricular myocytes of neonatal rats. Whole‐cell records of currents in 40 mmol/L Na+ outside (see inset) (Kunze et al. 1985). (B) Bovine single ventricular myocytes. Two microelectrode voltage clamp on single cells. The clamp step depolarized the cell from −50 mV to 0 mV. Measurement of L‐type calcium current and effect of adrenaline 0.2 *μ*mole (Isenberg and Klöckner 1982). Permission granted.

Since the introduction of the single cell combined with the patch clamp the study of ionic currents has been much facilitated. Undistorted recordings of the fast Na^+^ current (Fig. 24A) and the initial part of the Ca^2+^ current (Fig. 24B) were possible. Current–voltage relations for TTX‐sensitive Na^+^ current from single ventricular myocytes of neonatal rats and examples of currents obtained (see inset) illustrate the improved quality of the results (Kunze et al. 1985). The Ca^2+^ current in bovine single ventricular myocytes instead of showing only a “slow” current now consists of a fast starting deflection followed by a slow component (Isenberg and Klöckner 1982). In the following paragraphs I would like to summarily present an overview of the most important findings on ionic currents obtained in the course of the two last decades.

#### Sodium currents

The late sodium current (also called persistent current, slow current, slowly inactivating current) (Makielski 2016; Giles and Carmeliet 2016) extends the effect of the sodium current from the fast upstroke of the action potential and its role in conduction to the plateau phase and repolarization.

The first suggestion of the sodium current playing a role during the plateau of the action potential was the finding of a TTX‐induced shortening of the action potential in cardiac Purkinje fibers (Dudel et al. 1967b; Coraboeuf et al. 1979). This shortening effect was first explained as due to a block of the Na^+^ window current (Attwell et al. 1979). The underlying ionic current, however, showed time dependency (Carmeliet and Saikawa 1982). In the rabbit Purkinje fiber the current is declining during up to tens of seconds. Its kinetics are completely different from those of the peak Na^+^ current. Time constants of inactivation increase at more depolarized potentials and recovery is relatively fast (Carmeliet 1987). The current is sensitive to TTX and blocked by ranazoline. The late current plays an important role in prolonging the action potential duration and may cause early after depolarizations and ventricular arrhythmia. It also has been hypothesized to provoke intracellular Na^+^ overload and secondarily Ca^2+^ accumulation. An hypothetical “fuzzy space” (Lederer et al. 1990) would facilitate such phenomenon. This claim is far from proven and recently the hypothesis has been weakened by experiments on giant patches of guinea pig ventricle, which according to the analysis of Lu and Hilgemann (2017) strongly suggest that a fuzzy space with a restriction of free diffusion of Na^+^ ions does not exist in cardiac cells. Lu and Hilgemann conclude that measurements of “Na/K pump inactivation, subcellular Na, and cytoplasmic ion turnover kinetics contradict restricted Na spaces in murine cardiac myocytes.”

##### Neuronal Na^+^ channels expressed in cardiac cells

The presence of neuronal Na^+^ channel proteins in the T‐tubular system of cardiac cells was initially described by Maier et al. (2002, 2004).The following channel types have been found expressed: nNav1.1, nNav1.3, nNav1.6, of which nNav1.6 is the most abundant. They are characterized by a high sensitivity to TTX block in the nanomolar concentration scale, in comparison with the cNav1.5 channel of the surface membrane in the intercalated disks, which is inhibited by micromolar TTX concentrations. The typical cardiac Na^+^ channel, is responsible for an efficient conduction of the action potential. The neuronal channels in the T‐tubules play a role in the excitation–contraction coupling. They are responsible for the rapid depolarization which results in activation of the Ca^2+^current and elicit Ca^2+^ release occurring simultaneously at the periphery and the center of the cell. In cells without T‐tubules (atrial cells and Purkinje fibers) release of Ca^2+^ ions starts at the periphery and spreads afterward to the center (Hüser et al. 1996). In the presence of excessive catecholaminergic stimulation nNav channels can be phosphorylated and show an increased late Na^+^ current (Radwanski et al. 2016).

#### Ca^2+^ currents

I mentioned already the transformation of the Ca^2+^current from a slow to a fast type. A substantial part inactivates rapidly but the rest shows slow to very slow inactivation. This has provided the name of L‐type Ca^2+^ current; L stays for “long.” The inactivation process is voltage‐ and Cai^2+^‐dependent. Most of the Ca^2+^ responsible for the inactivation is coming from the SR (sarcoplasmatic reticulum). The Ca^2+^‐dependent inactivation is a fast phenomenon (first 50 msec) (Sipido et al. 1995), voltage‐dependent inactivation is much slower. The L‐type Ca^2+^ current is responsible for the excitation‐contraction coupling. Channel activity is enhanced by beta‐adrenergic stimulation and can be blocked by dihydropyridines, phenylalkylamines, and benzothiazepines (Tsien et al. 1986).

A so‐called T‐type Ca^2+^ current (T stands for “transient”) has been described by two groups in the same year 1985 (Bean 1985; Nilius et al. 1985). It has a short duration and is activated at potentials more negative than the threshold for L‐type channels. Activation and inactivation are rapid; inactivation is complete. It is blocked by mibefradil and insensitive to beta‐receptor stimulation (Tytgat et al. 1988). The channel plays an important role in pacemaking, as inward current carrier not only but also as inducer of Ca^2+^ sparks and secondarily activating the Na^+^,Ca^2+^ exchange transporter.

#### Positive dynamic current: follow‐up

In the preceding section of early voltage‐clamp results in cardiac cells the positive dynamic current was first described as a voltage‐activated Cl^−^ current, followed afterward as a Cai^2+^‐activated K^+^ current. With the improvement of the voltage‐clamp technique the current was split into two components: Ito1, a voltage‐activated K^+^ current and Ito2, a Cai^2+^‐activated current of unknown ionic nature. With the patch electrode and simultaneous fluorescence intracellular Ca^2+^ measurements on single cells of the rabbit Purkinje fiber evidence was presented that a Ca^2+^‐activated Cl^−^ current is responsible for the Ito2 current (Sipido et al. 1993). The circle is closed again; a similar current is also present (Zygmunt and Gibbons 1991; Zygmunt and Gibbons 1992) in rabbit ventricular and atrial myocytes. At the present time the existence of two components of the positive dynamic current has been confirmed in many cardiac preparations (Tseng and Hoffman 1989; Duran et al. 2010).

#### Potassium currents

##### Inward rectifier IK1

In the section on the reinvention of cardiac electrophysiology, IK1 was described as a time‐independent current, important and energy‐saving for the genesis of the long action potential. A transient accumulation of extracellular K^+^ was assumed to play a role during the rapid repolarization. In the period after the introduction of the patch electrode, the IK1 current has been found to undergo fast time‐dependent changes during the course of the action potential, due to block by Mg^2+^ ions on depolarization (Matsuda et al. 1987; Vandenberg 1987). Some years later putrescine, spermidine, and spermine were added as efficient blockers of IK1 (Lopatin et al. 1994). According to a study by Shimoni et al. (1992) using the action potential voltage clamp, IK1 is not only blocked but undergoes significant “inactivation” during the plateau. It exhibits time‐ and voltage‐dependent reactivation during repolarization as well as during early diastole. It is the primordial current during the final repolarization.

##### Ligand‐activated K^+^ currents

###### KATP channel

Hypoxia and application of metabolic inhibitors (DNP or CN) have been shown to shorten the cardiac action potential duration in single ventricular myocytes of the guinea pig (Isenberg et al. 1983). In the same study voltage clamp measurements demonstrated that this effect was due to an increase in time‐independent outward current. In the same year Noma (1983) demonstrated in guinea pig and rabbit atrial and ventricular cells the existence of an ATP‐dependent channel, which was activated by a fall of intracellular ATP concentration. Independently Trube G. and Hescheler J. (cited by Noma) reported also in 1983, a similar ATP‐dependent channel in guinea pig ventricular cells. A dose‐response curve obtained in inside‐out patch indicates a 50% activation at 0.1 mmol/L ATP. It is not activated by intracellular Ca^2+^ions. In the pancreas it is interacting with the beta cells to determine the maintenance of the glucose concentration in the blood between appropriate limits. In cardiac cells it shortens the action potential and eventually causes inexcitability.

###### ACH‐sensitive K^+^ current

The IKAch current is activated upon binding of Ach to the M2 muscarinic receptor, coupled to the channel via a Gi‐protein. The channel is very selective for K^+^ions. It is expressed in the sinoatrial node, the atria, the conducting system and in a number but not all ventricular cells. The current is weakly inward‐rectifying. Activation causes decrease in heart rate, hyperpolarization of the resting potential and shortening of the action potential (Heidbüchel et al. 1987).

#### Delayed K^+^ currents

In the early eighties repolarization in cardiac cells was explained by activation of a delayed slowly activating K^+^ current called IK. It was evident, however, that more than one current was involved. In 1990 (Sanguinetti and Jurkiewicz 1990) Sanguinetti and Jurkiewicz were able, using the sulfonamide drug E‐4031 to dissect the complex current into two units, IKr and IKs. IKr, blocked by E‐4031, is rapidly activated and rapidly inactivated. IKs shows slow activation and no inactivation. In fully activated state (long duration depolarizations) IKs is 10 times larger than IKr. For shorter depolarizations corresponding to the duration of the action potential, both currents, because of the difference in activation speed, are of similar amplitude. The IKr current shows a pronounced inward rectification. The underlying mechanism has been analyzed by Shibasaki (1987) and is due to fast inactivation preceding partly the activation process. The mechanism underlying the genesis of the inward rectification in IKr is thus different from the block by intracellular positive charges described for the IK1 channel.

A third member is the ultrapid delayed rectifier IKur, with no or very slow inactivation (Nerbonne, 2000). Recovery from inactivation is very slow; the current is markedly reduced at elevated frequencies. It is present in different cardiac preparations, including human atria and very sensitive to 4‐AP; it is increased by beta‐receptor stimulation and inhibited by alpha‐receptor activation.

### End

Bernstein has given us an explanation for the genesis of the resting membrane potential in excitable tissues. This thesis is still valid. His explanation for the action potential had to be changed when transmembrane potential measurements showed the existence of an overshoot and its dependence on external Na^+^ concentration, and shortly afterward by the detailed description of the conductance changes for Na^+^ and K^+^ ions in the squid giant axon. J.Z.Young's intuition was fortunate for electrophysiologic research when he, being an anatomist, convinced physiologists to use the squid giant axon in their investigations.

By writing “From Bernstein's rheotome to Neher–Sakmann's patch electrode” it was my intention to describe the evolution, first in nerve but later limited to heart, of facts and hypotheses on the genesis of the action potential.

I kept silent on the genesis of spontaneous activity and the propagation of the action potential. These two important topics will be treated in separate short reviews.

## Conflict of Interest

None declared.
